# Effect of Selective Personality-Targeted Alcohol Use Prevention on 7-Year Alcohol-Related Outcomes Among High-risk Adolescents: A Secondary Analysis of a Cluster Randomized Clinical Trial

**DOI:** 10.1001/jamanetworkopen.2022.42544

**Published:** 2022-11-17

**Authors:** Nicola C. Newton, Jennifer Debenham, Tim Slade, Anna Smout, Lucinda Grummitt, Matthew Sunderland, Emma L. Barrett, Katrina E. Champion, Cath Chapman, Erin Kelly, Siobhan Lawler, Natalie Castellanos-Ryan, Maree Teesson, Patricia J. Conrod, Lexine Stapinski

**Affiliations:** 1 The Matilda Centre for Research in Mental Health and Substance Use, The University of Sydney, Sydney, Australia; 2 Department of Psychiatry, University of Montreal, Montreal, Quebec, Canada; 3 Sainte Justine Hospital Research Centre, Montreal, Quebec, Canada

## Abstract

**Importance:**

Alcohol consumption is one of the leading preventable causes of burden of disease worldwide. Selective prevention of alcohol use can be effective in delaying the uptake and reducing harmful use of alcohol during the school years; however, little is known about the durability of these effects across the significant transition from early adolescence into late adolescence and early adulthood.

**Objective:**

To examine the sustained effects of a selective personality-targeted alcohol use prevention program on alcohol outcomes among adolescents who report high levels of 1 of 4 personality traits associated with substance use.

**Design, Setting, and Participants:**

A cluster randomized clinical trial was conducted to assess the effectiveness of the selective personality-targeted PreVenture program on reducing the growth of risky alcohol use and related harms from early to late adolescence and early adulthood. Participants included grade 8 students attending 14 secondary schools across New South Wales and Victoria, Australia, in 2012 who screened as having high levels of anxiety sensitivity, negative thinking, impulsivity, and/or sensation seeking. Schools were block randomized to either the PreVenture group (7 schools) or the control group (7 schools). The primary end point of the original trial was 2 years post baseline; the present study extends the follow-up period from July 1, 2017, to December 1, 2019, 7 years post baseline. Data were analyzed from July 22, 2021, to August 2, 2022.

**Interventions:**

The PreVenture program is a 2-session, personality-targeted intervention designed to upskill adolescents to better cope with their emotions and behaviors.

**Main Outcomes and Measures:**

Self-reported monthly binge drinking, alcohol-related harms, and hazardous alcohol use measured by the Alcohol Use Disorders Identification Test–Concise consumption screener.

**Results:**

Of 438 participants (249 male [56.8%]; mean [SD] age, 13.4 [0.5] years) from 14 schools, 377 (86.2%) provided follow-up data on at least 2 occasions, and among those eligible, 216 (54.0%) participated in the long-term follow-up. Compared with the control condition, the PreVenture intervention was associated with reduced odds of any alcohol-related harm (odds ratio [OR], 0.81 [95% CI, 0.70-0.94]) and a greater mean reduction in the frequency of alcohol-related harms (β = −0.22 [95% CI, −0.44 to −0.003]) at the 7.0-year follow-up. There were no differences in the odds of monthly binge drinking (OR, 0.80 [95% CI, 0.56-1.13]) or hazardous alcohol use (OR, 0.87 [95% CI, 0.59-1.27]) at the 7.0-year follow-up. Exploratory analyses at the 5.5-year follow-up showed that compared with the control condition, the PreVenture intervention was also associated with reduced odds of monthly binge drinking (OR, 0.87, [95% CI, 0.77-0.99]) and hazardous alcohol use (OR, 0.91 [95% CI, 0.84-0.99]), but this was not sustained.

**Conclusions and Relevance:**

This study demonstrated that a brief selective personality-targeted alcohol use prevention intervention delivered in the middle school years can have sustained effects into early adulthood.

**Trial Registration:**

anzctr.org.au Identifier: ACTRN12612000026820

## Key Points

**Question:**

What is the long-term effect of selective personality-targeted intervention on alcohol outcomes over 7 years among adolescents?

**Findings:**

In this secondary analysis of a cluster randomized clinical trial including 438 participants, the school-based PreVenture intervention was effective in reducing the odds of alcohol-related harms and the mean frequency with which alcohol-related harms were experienced, from early adolescence (aged 13.5 years) to early adulthood (aged 20.5 years), compared with controls.

**Meaning:**

These findings suggest that selective school-based prevention for alcohol misuse that provides coping skills relevant to individual personality types can produce favorable health effects that can be sustained into early adulthood.

## Teaser

This secondary analysis of a cluster randomized trial reports alcohol use outcomes from the PreVenture program at 5.5-year and 7.0-year assessments among young Australians who reported 1 of the 4 personality traits associated with substance use.

## Introduction

Alcohol use is one of the leading causes of preventable morbidity and mortality globally.^1^ In the US, excessive alcohol use is responsible for nearly 100 000 deaths and 2.7 million years of life lost every year.^2^ The burden of alcohol use is ubiquitous, extending to alcohol use disorder, suicide, chronic disease, poisonings, injuries, and traffic accidents.^1,2^ Effective prevention of alcohol misuse and alcohol use disorder is therefore critical to reduce the associated individual and societal burden. Evidence suggests that early initiation of alcohol use is associated with an increased risk of subsequent hazardous use and comorbid mental health problems.^3,4,5^ Indeed, the risk of developing alcohol use disorder is reduced by 9% for every year that onset is delayed.^6^ Thus, adolescence represents a critical period for the prevention of alcohol use and misuse.^7,8^

Several effective prevention approaches for adolescent alcohol misuse exist, among which school-based programs are common.^9^ School-based prevention programs typically adopt a universal approach, that is, they are delivered to the whole population regardless of their level of risk. These programs show small to moderate effect sizes in preventing or reducing alcohol use.^10^ Although trends during the last 20 years show encouraging reductions in rates of adolescent alcohol use, there remains a substantial proportion of adolescents who continue to consume alcohol at risky levels.^11,12,13^ For example, 14% of US 12th graders and 26% of Australians aged 16 to 17 years are binge drinkers (ie, consume ≥5 standard alcoholic drinks per day) fortnightly.^14,15^ This suggests the efficacy of prevention approaches may be improved by targeting those most at risk for alcohol use, who may respond to health education as normal.

Selective programs typically show larger effect sizes in preventing alcohol use compared with universal programs.^10,16^ One selective prevention program, PreVenture, targets adolescents exhibiting personality traits linked to an increased risk of alcohol misuse: hopelessness (ie, negative thinking), anxiety sensitivity, impulsivity, and sensation seeking.^17,18,19^ The program encourages young people to develop healthy coping skills to deal with personality-specific emotional and behavioral reactions before the onset of alcohol use, reducing the likelihood of using alcohol as a coping mechanism. The effectiveness of the PreVenture program in reducing and preventing alcohol use has been demonstrated through several randomized clinical trials in Europe, North America, and Australia.^18,20,21,22^ The Australian trial^22^ demonstrated that PreVenture successfully reduced growth in alcohol use, binge drinking, and alcohol-related harms from early to middle adolescence (13-16 years of age).

Whether the beneficial effects of school-based prevention endure into late adolescence and early adulthood is unknown. Few longitudinal studies to date have examined outcomes beyond 3 years,^23^ and to our knowledge no studies have examined the long-term sustainability of selective prevention.^24^ In Australia, late adolescence follows the legal age for alcohol purchase and is associated with a rapid escalation in use. Similarly, early adulthood represents a significant and often stressful life transition, when entering the workforce and beginning higher education are both associated with greater vulnerability to alcohol use disorder.^25^ Risky alcohol use causes sustained deficits in neurodevelopment—including structural, functional, and cognitive aberrations—until 25 years of age.^26^ Given that the peak age at onset of substance use disorders is 19.5 years, understanding the durability of school-based prevention around this period is critical.^25^

This study reports alcohol use outcomes from the PreVenture program at 5.5- and 7.0-year assessments among young Australians who reported high levels of 1 of the 4 personality traits associated with substance use, and represents the longest follow-up of a selective prevention program to date.^27^ A cluster randomized clinical trial was conducted to examine the effects of PreVenture on binge drinking, hazardous alcohol use, and alcohol-related harm among young people compared with a control group. We hypothesized that young people who received the PreVenture intervention would show reduced odds of all alcohol-related outcomes at the 5.5- and 7.0-year assessments compared with the control group.

## Methods

### Study Design

This study sample was derived from a 4-group cluster randomized clinical trial that began in 2012^28^ and had an original primary end point at 2 years post baseline. An extended follow-up of this trial saw data collection continue from July 1, 2017, to December 1, 2019 (7 years post baseline), across 7 assessments. Study protocols for the original and extended follow-up trials published elsewhere^28,29^ are provided in Supplement 1 and contain full details of the study design. The research protocol for the extended follow-up study^30^ was approved by the Human Research Ethics Committees of the University of New South Wales and The University of Sydney. Written informed consent was obtained from all students and 1 parent or caregiver to participate in the initial trial. Trial reporting follows the Consolidated Standards of Reporting Trials (CONSORT) guidelines.

The trial was designed to examine the long-term effectiveness of universal (delivered to all), selective (personality-targeted), and combined (universal and selective) school-based prevention programs targeting alcohol use and misuse and related harms. Cluster randomization was used with each school constituting 1 cluster to avoid contamination of the control group with the intervention group via student communication and to support equivalence of trial group sizes. An external researcher (T.S.) conducted block randomization using an online tool^31^ to randomly allocate participating Australian schools (N = 26) to 1 of 4 study conditions: (1) Climate Schools, with universal web-based prevention delivered to all students; (2) PreVenture, with selective personality-targeted prevention; (3) Climate Schools and PreVenture (CAP), with delivery of both the universal and selective programs; or (4) control, with drug and alcohol education as usual (approximately 2-10 lessons).

As specified in our protocol for the long-term follow-up,^29^ these a priori analyses will focus on students categorized as having high levels of 1 of 4 personality traits associated with substance use and investigate for the first time, to our knowledge, the long-term sustainability (7 years) of a selective prevention program compared with health education as usual. As per the study protocol,^29^ exploratory analyses were conducted to assess intervention effects at the 5.5-year assessment (mean [SD] age, 19.0 [0.4] years), given the critical timing of this assessment in capturing change from the 3.0-year assessment (mean [SD] age, 16.4 [0.4] years) to adulthood, following the legal age of alcohol purchase in Australia from 18 years.

### Participants

The intention-to-treat sample for this study consisted of 438 year 8 students (mean [SD] age, 13.4 [0.5] years) attending school in Australia in 2012. This was a subset of a larger trial involving 2190 students from 26 schools. The study focuses on students with elevated levels of any of 4 personality traits (anxiety sensitivity, negative thinking, impulsivity, and sensation seeking) in schools allocated to the PreVenture intervention (n = 7) or the active control condition (n = 7). Participants were asked which of the following best described their gender identity: male, female, nonbinary/gender fluid, or different. Race and ethnicity data were not specifically collected, although participants were asked about their country of birth.

### Procedure

All participants present at baseline, except those who withdrew consent, were eligible to take part in the long-term follow-up. Assessments involved online self-report surveys accessed via the study website.^32^ Confidentiality was ensured throughout the trial, and participant responses were linked over time using a unique identification code. Extensive retention procedures were implemented and outlined elsewhere.^33^ The Figure depicts the study flow and participant retention rates across all time points.

**Figure.**
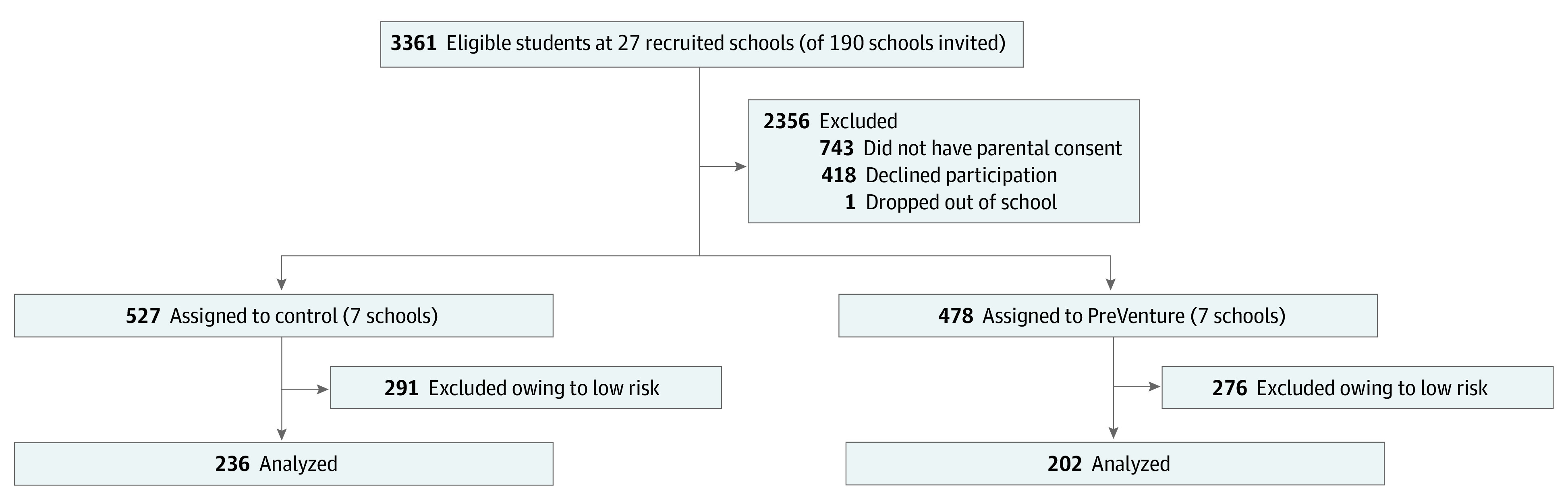
Study Flow Diagram

### Screening

Categorization of participants into personality groups was based on student responses to the Substance Use Risk Profile Scale (SURPS)^34^ at baseline. The SURPS is validated among Australian adolescents^35^ and consists of 23 self-report items measuring 4 well-validated personality risk factors for substance misuse: impulsivity, sensation seeking, anxiety sensitivity, and negative thinking.^34^ Students who scored 1 or more SDs above the school mean on any of the 4 personality risk subscales were categorized as having elevated levels of that trait.

### Intervention

PreVenture is a brief personality-targeted, selective intervention consisting of two 90-minute sessions delivered 1 week apart (a full description of the intervention is provided in the eMethods in Supplement 2). Sessions are conducted separately for and tailored to each personality group. For example, all participants who scored at least 1 SD above the mean on the Sensation Seeking subscale formed 1 group and received a version of PreVenture tailored to the sensation seeking risk profile. Students with elevated scores on more than 1 SURPS subscale (132 [30.1% of baseline sample]) were allocated to the personality group corresponding to their subscale score that deviated most from the mean, according to *z* scores. Sessions were delivered by trained facilitators in adherence to the training protocol described by O’Leary-Barrett et al.^36^ Facilitators were registered clinical psychologists (including N.C.N., E.L.B., K.E.C., and E.K.), and cofacilitators (L.G. and S.L.) were research assistants with Bachelor of Psychology (Honors) qualifications.

The 2 sessions incorporated psychoeducation, motivation enhancement therapy, and cognitive behavioral therapy components into clinical situations to explore helpful and unhelpful coping strategies common to each personality profile and goal-setting exercises. Students were introduced to the cognitive behavioral model through examination of their emotional, behavioral, and physiological reactions to a recent experience and then were supported to identify and challenge profile-specific cognitions that precede problematic behaviors. No additional booster doses of the intervention were delivered.

### Measures

The developmentally appropriate outcomes of interest were monthly binge drinking, occurrence of any alcohol-related harm, and hazardous alcohol use. Binge drinking was assessed using the Patterns of Alcohol Index.^37^ Participants rated the frequency of their binge drinking, that is, consuming 5 or more standard drinks (10 g of alcohol) in a single occasion within the past 6 months. Ratings were made on a 6-point Likert scale ranging from “never” to “daily or almost daily,” where responses were dichotomized into monthly or more occurrences of binge drinking (yes or no). Monthly binge drinking was chosen as the cutoff to align with normative levels of binge drinking in emerging adult populations. In Australia, 41% of those aged 18 to 24 years binge drink on at least a monthly basis.^38^

Alcohol-related harms were assessed using a 9-item version of the Rutgers Alcohol Problem Index,^39^ a self-report questionnaire measuring problem alcohol consumption in adolescence. Participants rated the frequency with which they experienced adverse outcomes of alcohol use (eg, “got into fights, acted bad or did mean things” or “neglected my responsibilities”) on a 5-point Likert scale ranging from “never” to “more than 6 times” within the previous 6 months. Item responses were dichotomized and used to create a summary item representing the presence of any alcohol-related harms in the past 6 months. In addition, a total score for the frequency of alcohol-related harms was calculated by summing the frequency of each of the 9 items endorsed by participants, where “never” is scored 0 and “more than 6 times” is scored 4 to produce a possible total score of 36.

Hazardous alcohol use was assessed by combining items measuring (1) frequency and quantity of alcohol consumption and (2) frequency of binge drinking. This score is equivalent to that derived from the Alcohol Use Disorders Identification Test–Concise (AUDIT-C) alcohol consumption screener.^40^ In alignment with established AUDIT-C cutoff scores, scores of 3 or more were interpreted as indicative of hazardous alcohol use and possible alcohol use disorder.^41^ Hazardous alcohol use was measured at all assessment occasions throughout the trial to assess emerging symptoms of alcohol use disorder and is presented in place of the *Diagnostic and Statistical Manual of Mental Disorders, Fifth Edition* self-report symptom, which was only assessed at the long-term follow-up.

### Statistical Analysis

#### Modeling Approach

Multilevel mixed-effect regression models^42^ were used to determine the long-term effects of the intervention at the 5.5- and 7.0-year assessments. Intervention effects were measured by the group × time interaction, and the best-fitting model for time was selected representing growth from baseline to the 0.5-, 1.0-, 2.0-, 3.0-, 5.5-, and 7.0-year assessments. Alcohol-related harm (yes or no), the frequency of alcohol-related harms (total Rutgers Alcohol Problem Index score), and hazardous alcohol use (yes or no) exhibited linear growth patterns, and monthly binge drinking (yes or no) exhibited a quadratic growth pattern, where time represents the relative 1-year change in odds of the outcome. The interaction term for binary outcomes (monthly binge drinking, alcohol-related harms, and hazardous alcohol use) were modeled via the logit link function, and the odds were calculated by exponentiating the regression coefficient. The continuous outcome for the frequency of alcohol-related harms was modeled via a multilevel negative binomial regression, which is a generalized Poisson regression that accounts for overdispersed count outcome variables.^43^ The discrete model-based estimates of each outcome at the 7.0-year follow-up were presented as odds for each binary outcome and as an incidence rate ratio for the continuous outcome. The best-fitting random-effects structure for each outcome was tested using likelihood ratio tests, with Akaike information criterion statistics to confirm the covariance structure.^44^ All models included both a random intercept at the individual (level 2) and school (level 3) levels, and a random slope at the individual level was included when it improved the model fit. Full-information maximum-likelihood procedures were used to handle missing data, whereby all available data (n = 438) are included in the model estimates under the assumption that data were missing at random. Multiple imputation was used to explore the impact of missing data on the robustness of the findings. For each outcome, 20 imputed data sets were created. Covariates associated with either missingness or the outcomes were included in the imputation model, including sex, baseline SURPS scores, and baseline alcohol-related outcomes. Sensitivity analyses were conducted to adjust for baseline group differences in sex. Descriptive statistics were run on baseline demographic data, and all analyses were conducted on the intention-to-treat sample using Stata, version 17 (StataCorp LLC).^45^ Data were analyzed from July 22, 2021, to August 2, 2022. Two-sided *P* < .05 indicated statistical significance.

## Results

### Baseline Characteristics, Outcome Frequency, and Attrition Across Follow-up

From a total of 1005 participants, 438 from 14 schools with elevated levels of 1 of the 4 personality traits completed the baseline survey (249 male [56.8%] and 188 female [42.9%], with data missing for 1 [0.2%]). The mean (SD) age of the sample at baseline was 13.4 (0.5) years; 373 participants (85.2%) were born in Australia and 339 (77.4%) attended a private school. There were more male participants in the PreVenture group (164 of 202 [81.2%]) than the control group (85 of 236 [36.0%]); baseline characteristics are provided in Table 1. Raw frequencies and mean prevalence of outcomes over time are provided in Table 2, with steady growth in outcomes occurring during the first 3 years of follow-up (mean [SD] ages, 13.4 [0.5] to 16.4 [0.4] years) followed by a steep escalation in use from the 3.0- to 5.5-year follow-ups (mean [SD] ages, 16.4 [0.4] to 19.0 [0.4] years) and slowed growth at the 7.0-year follow-up (mean [SD] age, 20.5 [0.5] years). Graphical representations of alcohol outcomes at all assessment occasions (Table 2) are provided in eAppendix 1 in Supplement 2. Missing data occurred owing to participants being absent from school, moving schools, failing to use their unique identifying code, or declining to take part, culminating in 417 participants (95.2% of eligible long-term follow-up sample) present at the long-term follow-up. Most participants completed at least 1 follow-up, 377 (86.2%) completed at least 2 follow-ups, and 216 (54.0%) of eligible participants participated in the long-term follow-up. The final models used all data from all participants (n = 438). Comparisons between those absent and present at long-term follow-up are presented in eAppendix 2 in Supplement 2. Students missing at the long-term follow-up were more likely to be male (OR, 1.80 [95% CI, 1.23-2.67]) and in the PreVenture group (OR, 1.35 [95% CI, 1.11-1.64]). Higher attrition in the PreVenture group was likely caused by the higher number of boys present at baseline (81.2% compared with 36.0%). None of the personality types or alcohol outcomes were associated with attrition at follow-up.

**Table 1.** Baseline Characteristics of the Sample

Characteristic	Participant group^a^	
	Control (n = 236)	PreVenture (n = 202)
No. of schools	7	7
Age, mean (SD), y	13.4 (0.5)	13.4 (0.5)
Sex		
Male	85 (36.0)	164 (81.2)
Female	150 (63.6)	38 (18.8)
Missing	1 (0.4)	0
Personality profile^b^		
Sensation seeking	61 (25.8)	57 (28.2)
Impulsivity	64 (27.1)	48 (23.8)
Anxiety sensitivity	58 (24.6)	59 (29.2)
Negative thinking	53 (22.5)	38 (18.8)
Country of birth		
Australia	206 (87.3)	167 (82.7)
Other English-speaking	15 (6.4)	18 (8.9)
Non–English-speaking	14 (5.9)	16 (7.9)
Missing	1 (0.4)	1 (0.5)
Private school	194 (82.2)	145 (71.8)

^a^ Unless otherwise indicated, data are expressed as No. (%) of participants.

^b^ Personality profiles were determined according to established cutoffs for the Substance Use Risk Profile Scale.

**Table 2.** Alcohol Outcomes at All Assessment Occasions

Outcome by assessment time	Participant group^a^	
	Control	PreVenture
Monthly binge drinking in past 6 mo (≥5 standard drinks on 1 occasion)		
Baseline	3/235 (1.3)	9/198 (4.5)
0.5 y	6/195 (3.1)	12/142 (8.5)
1.0 y	8/203 (3.9)	7/132 (5.3)
2.0 y	24/184 (13.0)	10/124 (8.1)
3.0 y	32/173 (18.5)	20/105 (19.0)
5.5 y	73/121 (60.3)	41/71 (57.7)
7.0 y	59/106 (55.7)	43/62 (69.4)
Any alcohol-related harms in the past 6 mo		
Baseline	26/235 (11.1)	44/198 (22.2)
0.5 y	26/195 (13.3)	30/132 (22.7)
1.0 y	38/203 (18.7)	28/132 (21.2)
2.0 y	59/185 (31.9)	42/124 (33.9)
3.0 y	81/173 (46.8)	49/105 (46.7)
5.5 y	102/121 (84.3)	55/70 (78.6)
7.0 y	87/105 (82.9)	47/62 (75.8)
Frequency of alcohol-related harms, mean (SD)^b^		
Baseline	3.99 (5.90)	6.77 (6.84)
0.5 y	2.92 (4.77)	6.34 (7.28)
1.0 y	2.38 (4.23)	4.68 (6.54)
2.0 y	2.55 (3.98)	3.90 (6.16)
3.0 y	2.91 (4.77)	4.04 (6.69)
5.5 y	4.23 (4.18)	3.95 (4.51)
7.0 y	3.61 (3.96)	3.49 (4.32)
AUDIT-C hazardous drinking (scores >3)		
Baseline	7/235 (3.0)	11/198 (5.6)
0.5 y	11/195 (5.6)	15/132 (11.4)
1.0 y	18/203 (8.9)	9/131 (6.9)
2.0 y	39/188 (20.7)	30/124 (24.2)
3.0 y	59/173 (34.1)	34/105 (32.4)
5.5 y	103/121 (85.1)	56/71 (78.9)
7.0 y	88/106 (83.0)	52/62 (83.9)

Abbreviations: AUDIT-C, Alcohol Use Disorders Identification Test–Concise.

^a^ Unless otherwise indicated, data are expressed as No./total No. (%) of participants.

^b^ Scored on a 6-point Likert scale ranging from “never” (1) to “daily or almost daily” (6).

### Intervention Effects

#### Monthly Binge Drinking

There was no evidence of a difference in the odds of monthly binge drinking between the PreVenture and control groups during the 7.0-year follow-up, when mean participant age was 13.5 to 20.5 years, for the linear growth component (OR, 0.80 [95% CI, 0.56-1.13]), multiple imputation (OR, 1.00 [95% CI, 0.93-1.15]), or quadratic growth component (OR, 1.03 [95% CI, 0.98-1.08]) of the model, which is consistent with the point estimate at 7 years (OR, 0.20 [95% CI, 0.02-2.31]). eAppendix 3 in Supplement 2 provides an exploratory analysis of monthly binge drinking during the 5.5-year follow-up when mean participant age was 13.5 to 19.0 years.

#### Alcohol-Related Harm

Relative to the control condition, the PreVenture intervention was associated with a sustained reduction in the odds of experiencing any alcohol-related harm during the 7.0-year follow-up (OR, 0.81 [95% CI, 0.70-0.94]; OR for multiple imputation, 0.30 [95% CI, 0.06-0.77]) (Table 3). This finding represents an annual 19% odds reduction of reporting alcohol-related harms between 13.5 to 20.5 years of age and is consistent with the point estimate at the 7.0-year assessment (OR, 0.23 [95% CI, 0.08-0.63]). Furthermore, relative to the control condition, the PreVenture intervention was associated with a greater mean reduction in the frequency of alcohol-related harms experienced during the 7.0-year follow-up (β = −0.22 [95% CI, −0.44 to −0.003]; multiple imputation β = −0.16 [95% CI, −0.23 to −0.09]). This is consistent with the point-estimate at 7 years (incidence rate ratio, 0.20 [95% CI, 0.04-0.98]).

**Table 3.** Relative Annual Change in Odds, Mean Group × Time Interactions, and Point-Specific Estimates at the 7-Year Assessment for the PreVenture vs Control Groups

	Change data (95% CI)	*P* value
Monthly binge drinking, OR		
PreVenture × time	0.80 (0.56 to 1.13)	.20
PreVenture × time squared	1.03 (0.98 to 1.08)	.26
PreVenture vs control at 7.0 y	0.20 (0.02 to 2.31)	.20
Any alcohol-related harm, OR		
PreVenture × time	0.81 (0.70 to 0.94)	.004
PreVenture vs control at 7.0 y	0.23 (0.08 to 0.63)	.004
Hazardous alcohol use (AUDIT-C), OR		
PreVenture × time	0.87 (0.59 to 1.27)	.47
PreVenture vs control at 7.0 y	0.37 (0.03 to 5.22)	.47
Frequency of alcohol-related harms, β	−0.22 (−0.44 to −0.003)	.05
PreVenture × time		
PreVenture vs control at 7.0 y, IRR	0.20 (0.04 to 0.98)	.05

Abbreviations: AUDIT-C, Alcohol Use Disorders Identification Test–Concise; IRR, incidence rate ratio; OR, odds ratio.

#### Hazardous Alcohol Consumption

There was no evidence of a difference in the odds of hazardous alcohol use at the 7.0-year follow-up between the PreVenture and control groups (OR, 0.87 [95% CI, 0.59-1.27]; OR for multiple imputation, 0.92 [95% CI, 0.80-1.05]), which is consistent with the point estimate at the 7.0-year follow-up (OR, 0.37 [95% CI, 0.03-5.22]). eAppendix 3 in Supplement 2 provides an exploratory analysis of hazardous alcohol consumption at the 5.5-year follow-up. The results from the multiple imputation analysis confirm the robustness of the findings that the PreVenture intervention reduced the odds of experiencing any alcohol-related harms and the total mean frequency of alcohol-related harm over time, compared with the control condition (Table 4).

**Table 4.** Relative Change in Odds and Mean Group × Time Interactions of Alcohol Outcomes During 7-Year Study Period for the PreVenture vs Control Groups Using Multiple Imputation

Alcohol outcome	Change data (95% CI)	*P* value
Monthly binge drinking, OR		
PreVenture × time	1.00 (0.93 to 1.15)	.94
PreVenture × time squared	1.08 (0.92 to 1.23)	.83
Any alcohol-related harm, OR		
PreVenture × time	0.30 (0.06 to 0.77)	.01
Hazardous alcohol use (AUDIT-C), OR		
PreVenture × time	0.92 (0.80 to 1.05)	.24
Frequency of alcohol-related harms, β coefficient		
PreVenture × time	−0.16 (−0.23 to −0.09)	.002

Abbreviations: AUDIT-C, Alcohol Use Disorders Identification Test–Concise; OR, odds ratio.

### Sensitivity Analysis

To account for baseline differences in sex, sex was included as a covariate in the models, and adjusted estimates are presented in eAppendix 3, Table 2, in Supplement 2. Results showed negligible differences in parameter estimates, with the regression coefficients and 95% CIs remaining relatively unchanged. In addition, sensitivity analyses examined intervention effects at the 5.5-year assessment, given the critical timing of this assessment in capturing change from preadulthood (3.0-year assessment at a mean age of 16.5 years) to adulthood and legal alcohol purchase age (mean age, 19.5 years). The models omitting the 7.0-year survey assessment are presented in eAppendix 3, Table 3, in Supplement 2. Results at the 5.5-year follow-up demonstrated that the PreVenture intervention was also associated with reduced odds of monthly binge drinking (OR, 0.87 [95% CI, 0.77-0.99]) and hazardous alcohol use (OR, 0.91 [95% CI, 0.84-0.99]) compared with the control condition.

## Discussion

To our knowledge, this cluster randomized clinical trial is the first to demonstrate that selective personality-targeted alcohol use prevention delivered in middle school can have positive sustained effects over a 7-year period, from late adolescence to early adulthood. Robust analyses showed that compared with a control condition, participants who received a brief PreVenture intervention at a mean age of 13.5 years had reduced odds of reporting any alcohol-related harm and a significant mean reduction in the frequency of alcohol-related harms experienced over the 7-year study period from adolescence to young adulthood (mean age, 20.5 years). Notably, the findings of an exploratory analysis suggest that PreVenture was effective in reducing the annual odds of monthly binge drinking and hazardous alcohol use from the 5.5-year follow-up until late adolescence (mean age, 19.0 years); however, this was not sustained into early adulthood. This novel finding may indicate a weakening association between rates of use and rates of harm as individuals transition from adolescence to young adulthood. Coinciding with this developmental transition are higher rates of normative alcohol use, which in Australia aligns with the legal alcohol purchase age of 18 years. In the Australian context, 9% of adolescents aged 14 to 17 years have engaged in past-month binge drinking, compared with 41% of young adults aged 18 to 24 years.^38^ Despite early adolescent alcohol use being a strong and consistent indicator of later dependent use and harm, the current findings indicate there may be an uncoupling of this association in older adolescence that requires further investigation. Nonetheless, PreVenture’s 7-year efficacy in reducing the mean frequency of adverse alcohol-related consequences in young adults vulnerable to risky substance use is an important finding that confirms the efficacy of this program in reducing the burden of substance use felt by the individual, community, and economy. A future study may include a longer-term follow-up past this normative period of alcohol use to investigate whether trends diverge again as participants transition further into adulthood.

This study adds to the evidence base for selective, personality-targeted prevention of alcohol use^16,22^ and, to our knowledge, represents the longest follow-up of PreVenture to date. The durability of prevention effects highlights PreVenture’s success in providing useful and relevant skills for young people. The focus on practical skill development differs from other interventions, which aim to increase knowledge or build self-efficacy to resist peer pressure,^9^ and holds promise for future intervention development. Whether long-term follow-up outcomes hold constant across the 4 SURPS personality profiles is unknown and offers an interesting direction for future research.

### Limitations

This study has some limitations. First, despite comprehensive retention efforts, missing data in the long-term follow-up were high, likely due to the 7-year length of the trial. Nonetheless, 53.7% participation at long-term follow-up is comparable to similar studies tracking young people during the transition into adulthood.^46,47^ Moreover, the intention-to-treat approach combined with the full-information maximum-likelihood estimations are expected to provide nonbiased parameter estimates. In addition, multiple imputations showed that alcohol-related harm results are robust against the impact of missing data, although binge drinking and hazardous alcohol use may be less robust. Attrition analyses demonstrated that only sex and trial group were associated with missingness at follow-up, with trial group likely driven by the baseline sex differences between the trial groups. Notably, none of the outcome variables or personality traits were associated with missingness at the long-term follow-up, suggesting the sample was unbiased toward these outcomes. Sensitivity analyses adjusting for sex (eTables 2 and 3 in Supplement 2) yielded results consistent with the unadjusted analyses, further supporting the generalizability of the results. Second, the self-report nature of the measures may be subject to social desirability bias. However, substance-related reporting among young people has indicated excellent discriminant^48^ and predictive^49^ validity, and 2 validated screening items were used to assess data integrity,^50^ which revealed, for example, at the 5.5 year follow-up, 185 participants (94.4%) responded truthfully. Additionally, the sample was predominately Australian born and English speaking, which is similar to the general Australian population^51^; however, future studies should focus on more diverse populations.

## Conclusions

This large-scale, long-term study spanning 7 years is the first, to our knowledge, to evaluate selective personality-targeted prevention among young adolescents who report 1 of 4 personality traits. The findings show that selective alcohol use prevention delivered in early adolescence can have sustained effects across the critical transition into late adolescence and early adulthood, particularly in reducing alcohol-related harms. By encouraging young people to develop personality-specific coping skills that can be applied across different situations, rather than focusing on alcohol use alone, the PreVenture program offers a scalable solution to reduce alcohol-related harms to 20 years of age. These long-lasting effects highlight the importance of delivering evidence-based prevention in schools and underscores the need for continued investment in school-based preventive initiatives.

## Notes

Supplement 1.
Trial Protocol and Ethics Application Form

Supplement 2. **eMethods.** Intervention Description **eAppendix 1.** Graphical Representations of Results **eFigure 1.** Monthly Binge Drinking in Past 6 Months (≥5 Standard Drinks on 1 Occasion) **eFigure 2.** Any Alcohol-Related Harms in the Past 6 Months **eFigure 3.** Cumulative Alcohol-Related Harms **eFigure 4.** AUDIT-C Hazardous Drinking Scores (>3) **eAppendix 2.** Attrition Analysis **eTable 1.** Baseline Differences Between Those Lost to Follow-up and Those Present at the Long-term Follow-up **eAppendix 3.** Sensitivity Analyses **eTable 2.** Relative Change in Log Odds of Alcohol Outcomes at 7 Years Post Baseline (Adjusted for Baseline Differences in Sex) **eTable 3.** Relative Change in Odds and Mean Frequency of Alcohol Outcomes and Mean Frequency of Alcohol Harms From Baseline to 5.5 Years

Supplement 3.
Data Sharing Statement
