# Supplementary material for: Effect of Selective Personality-Targeted Alcohol Use Prevention on 7-Year Alcohol-Related Outcomes Among High-risk Adolescents: A Secondary Analysis of a Cluster Randomized Clinical Trial
Source: JAMA Netw Open. 2022 Nov 17;5(11):e2242544. doi: 10.1001/jamanetworkopen.2022.42544 (PMC9672969; doi:10.1001/jamanetworkopen.2022.42544)
Supplement: Supplement 1. — Trial Protocol and Ethics Application Form [file jamanetwopen-e2242544-s001.pdf]

CLINICAL TRIAL PROTOCOL

A comprehensive universal and targeted intervention to prevent substance use and related harms in Australian adolescents: The CAP study

PREVENTING SUBSTANCE USE AND RELATED HARMS IN ADOLESCENTS

This study is funded by the National Health and Medical Research Council (App 1004744). This protocol was created by Emma Barrett and is based on the International Conference on Harmonisation (ICH) Guidelines for Good Clinical Practice (http://ichgcp.net/).

General Information

Sponsor:
National Health and Medical Research Council (NHMRC)
GPO Box 1421
Canberra ACT 2601
Phone: 13 000 64672
Email: nhmrc@nhmrc.gov.au

Investigators:
Prof Maree Teesson (Ph: 9385 0233)
Dr Patricia Conrod
Dr Nicola Newton (Ph: 9385 0159)
Dr Tim Slade

Associate Investigators:
Prof Gavin Andrews
Prof Ron Rapee
Dr Nickolai Titov

Project Coordinator:
Ms Emma Barrett (Ph: 9385 0164)

Trial site:
National Drug and Alcohol Research Centre (NDARC)
University of NSW
Sydney, NSW 2052
Ph: 9385 0333

Table of Contents

Background Information **6**

Name and description of the investigational product(s) 6

A summary of findings from nonclinical studies that potentially have clinical significance and from clinical trials that are relevant to the trial 7

Summary of the known and potential risks and benefits, if any, to human subjects 10

Description of and justification for the route of administration, dosage, dosage regimen, and treatment period(s) 10

A statement that the trial will be conducted in compliance with the protocol, GCP and the applicable regulatory requirement(s) 11

Description of the population to be studied 11

References to literature and data that are relevant to the trial, and that provide background for the trial 11

Trial Objectives and Purpose **15**

Detailed description of the objectives and the purpose of the trial 15

Trial Design **16**

A specific statement of the primary endpoints and the secondary endpoints, if any, to be measured during the trial 16

A description of the type/design of trial to be conducted (e.g. double-blind, placebo-controlled, parallel design) and a schematic diagram of trial design, procedures and stages 16

A description of the measures taken to minimize/avoid bias, including: (a) Randomization. (b) Blinding. 17

A description of the trial treatment(s) and the dosage and dosage regimen of the investigational product(s). Also include a description of the dosage form, packaging, and labelling of the investigational product(s) 18

The expected duration of subject participation, and a description of the sequence and duration of all trial periods, including follow-up, if any. 19

A description of the "stopping rules" or "discontinuation criteria" for individual subjects, parts of trial and entire trial 19

Accountability procedures for the investigational product(s), including the placebo(s) and comparator(s), if any 19

Maintenance of trial treatment randomization codes and procedures for breaking codes 20

The identification of any data to be recorded directly on the Case Report Forms (i.e. no prior written or electronic record of data), and to be considered to be source data 20

Selection and Withdrawal of Subjects 21

Subject inclusion criteria..21

Subject exclusion criteria...21

Subject withdrawal criteria...21

Treatment of Subjects 22

The treatment(s) to be administered 22

Medication(s)/treatment(s) permitted (including rescue medication) and not permitted before and/or during the trial 23

Procedures for monitoring subject compliance 23

Assessment of Efficacy..24

Specification of the efficacy parameters 24

Methods and timing for assessing, recording, and analysing of efficacy parameters 25

Assessment of Safety..26

Specification of safety parameters 26

The methods and timing for assessing, recording, and analysing safety parameters 26

Procedures for eliciting reports of and for recording and reporting adverse event and intercurrent illnesses 26

The type and duration of the follow-up of subjects after adverse events.......................................26

Statistics..27

A description of the statistical methods to be employed, including timing of any planned interim analysis(ses) 27

The number of subjects planned to be enrolled 27

The level of significance to be used...28

Criteria for the termination of the trial 28

Procedure for accounting for missing, unused, and spurious data...28

Procedures for reporting any deviation(s) from the original statistical plan 28

The selection of subjects to be included in the analyses 28

Direct Access to Source Data/Documents...29

Quality Control and Quality Assurance...32

Ethics 33

Data Handling and Record Keeping 34

Financing and Insurance 35

Publication Policy 36

Background Information

Name and description of the investigational product(s)

The *CAP (Climate and Preventure) intervention* is a comprehensive, school-based substance use prevention intervention. The CAP intervention combines the effective 'universal' *Climate Schools* program and the 'targeted' *Preventure* program with the aim of reducing substance use and related harms among adolescents.

The Climate Schools program:
The *Climate Schools* program aims to reduce the use of Australia's most commonly used licit and illicit drugs: alcohol and cannabis, and related harms. The program was pioneered by CI Teesson, CI Newton and AI Andrews and is based on the effective harm-minimisation approach to prevention (Newton et al., 2009a; 2009b; 2009c; McBride et al., 2006; Vogl et al., 2009). The *Climate Schools* program consists of twelve 40-minute lessons and is designed to fit within the school health curriculum. It is facilitated by the internet which guarantees complete and consistent delivery whilst ensuring high implementation fidelity. It uses cartoon storylines to engage and maintain student interest and involvement over time (Schinke et al., 2004). The first six lessons focus specifically on alcohol and are delivered approximately six months prior to the remaining six lessons which focus on cannabis. The first part of each lesson is completed individually over the internet where students are engaged through cartoon storylines which impart information about the short- and long-term effects of alcohol and cannabis, normative alcohol and cannabis use, refusal skills, and first aid. Students are provided with confidential login details to access the *Climate* website. The second part of each lesson is a group or class activity delivered by the teacher which reinforces the information in the cartoons and allows interactive communication between students. Teachers are provided with a manual containing the activities, implementation guidelines, links to the education syllabus and summaries for each lesson.(To view the *Climate Schools* program, visit www.climateschools.com and logon using the Username: 'Teacher' and Password: 'London').

The Preventure program:
The *Preventure* program is a brief personality-targeted substance use preventive intervention for high-risk adolescents aged 14-15 years. Unlike universal programs, this selected personality-targeted approach addresses four personality risk-factors for early-onset substance misuse and other risky behaviours: Sensation Seeking, Impulsivity, Anxiety Sensitivity and Hoplessness (Woicik et al., 2009). The *Preventure* program is also consistent with new models which conceptualise substance use as being driven by personality traits such as impulsivity and disinhibition (Dick et al., 2008).This program involves two 90-minute group sessions which are carried out by a trained facilitator and co-facilitator. The interventions are conducted using manuals which incorporate psycho-educational and cognitive behavioural components, and include real life scenarios shared by high-risk youth in specifically-organised focus groups. In the first session, participants are guided in a goal setting exercise, designed to enhance motivation to change behaviour. Psycho-educational strategies are used to teach participants about their target personality variable and associated problematic coping behaviours like avoidance, interpersonal dependence, aggression, risky behaviours and substance misuse. They are then introduced to the

cognitive behavioural model and guided in breaking down personal experience according to the physical, cognitive and behavioural components of an emotional response. A novel component to this intervention approach is the fact that all exercises discuss thoughts, emotions and behaviours in a personality-specific way, e.g. identifying situational triggers and cognitive distortions related to Sensation Seeking specifically. In the second session, participants are encouraged to identify and challenge personality-specific cognitive distortions that lead to problematic behaviours. *Preventure* is the first and only targeted school-based alcohol and illicit drug prevention programme that has been shown to prevent growth in alcohol and substance misuse in Canada and the UK (Conrod et al., 2006; 2008; 2010).

A summary of findings from nonclinical studies that potentially have clinical significance and from clinical trials that are relevant to the trial

Alcohol and other drug use is common in Australia and the burden of disease, social costs, and disability associated with this use is considerable (1, 9, 10). The peak of this disability occurs in those aged 15-24 years and corresponds with the typical age of initiation of alcohol and drug use (11). The high prevalence of substance use amongst adolescents is of particular concern given that early initiation to drug use is a risk factor for the development of substance use disorders, comorbid mental health problems, juvenile offending, impaired educational performance and early school dropout, all of which negatively impact on both current functioning and future life options (12-15).

To reduce the occurrence and cost of such problems, prevention is essential and needs to be initiated early before harmful patterns of drug use are established and begin to cause disability (16, 17). Although an array of school-based prevention programs exist (18-23), the majority show minimal effects in reducing drug use and related harms (24-27), and some have even reported iatrogenic effects (28). The most common factors which impede on effectiveness are; the focus on abstinence-based outcomes (29, 30), and implementation failure (31-33).

There are two common approaches to school-based drug education; 'universal' and 'targeted' (34). The targeted approach involves delivering programs which target specific populations, such as individuals at greatest risk for developing substance use problems. On the other hand, the universal approach aims to deliver interventions to all students regardless of their level of risk for drug use, and focus largely on teaching drug resistance skills (35). The major advantage of this approach is that it has the ability to reduce substance use on a larger scale (36, 37). Regardless of the approach, programs which have demonstrated the most success in increasing drug related knowledge, decreasing pro-drug attitudes and decreasing drug use behavior, are those programs which have a harm-minimization goal, are implemented correctly and use interactive delivery techniques (20, 38-40).

Given that schoolbased drug prevention is the primary means by which drug education is delivered, it is essential to focus on increasing program efficacy. Ideally, preventive interventions should aim to delay onset in both adolescents with low-risk profiles who may

be influenced to take up substances due to peer influence and social conformity, and adolescents with high-risk profiles whose underlying vulnerability to psychopathology can lead to substance misuse. Yet, there appear to be no models of well implemented programs that do this. The current proposal addresses this gap by developing and evaluating a comprehensive approach to preventing substance use and related harms in adolescents by combining effective 'universal' and 'targeted' school-based prevention programs.

The universal 'Climate' program:

The universal *Climate* program aims to reduce the use of Australia's most commonly used licit and illicit drugs: alcohol and cannabis (1). The *Climate* program which was pioneered by CI Teesson, CI Newton and AI Andrews is based on the effective harm-minimisation approach to prevention (2-4, 41, 42) and uses cartoon storylines to engage and maintain student interest and involvement over time (43). The program is facilitated by the internet which guarantees complete and consistent delivery whilst ensuring high implementation fidelity. The program is designed to fit within the school health curriculum and be implemented in Year 9 before significant exposure to alcohol and drug use occurs. The *Climate* program consists of twelve 40-minute lessons aimed at reducing the use of alcohol and cannabis and related harms. The first six lessons focus specifically on alcohol and are delivered approximately six month prior to the remaining six lessons which focus on cannabis. The first part of each lesson is completed individually over the internet where students are engaged through cartoon storylines which impart information about the short- and long-term effects of alcohol and cannabis, normative alcohol and cannabis use, refusal skills, and first aid. Students are provided with confidential login details to access the *Climate* website. The second part of each lesson is a group or class activity delivered by the teacher which reinforces the information in the cartoons and allows interactive communication between students. Teachers are provided with a manual containing the activities, implementation guidelines, links to the education syllabus and summaries for each lesson.

The efficacy of the *Climate* program has been established using a cluster RCT in 10 schools in Sydney, Australia (n = 764) (2, 3). Results of the trial demonstrated that compared to the control group, students in the intervention group showed significant improvements in alcohol and cannabis knowledge at end of the course and at six and twelve months following the intervention. In terms of behavior change, the intervention group showed a reduction in frequency of cannabis use at the six- month follow-up, a reduction in average weekly alcohol consumption at the six and twelve month follow-ups, and a reduction in frequency of drinking to excess twelve months following the intervention. In addition, program evaluation showed that students and teachers rated the program as an acceptable and enjoyable means of delivering drug education in schools. Specifically, 100% of teachers who implemented this program in their classroom rated it as superior to other drug prevention programs, and over 90% of students reported information delivered in this format was easy to learn and would like more school subjects to be taught through this method.

Despite these positive results, the effectiveness of the universal *Climate* program is limited. Firstly, it is intended only to reduce the use of alcohol and cannabis and not other substances. As the prevalence of illicit drug use other than cannabis is relatively low

amongst adolescents, it has been suggested that such drugs may be better addressed using targeted rather than universal prevention programs (44). Secondly, like most universal prevention programs, *Climate* does not presume to remedy youth already abusing substances or those at high-risk of developing substance use disorders due to underlying vulnerability. This, along with recent research in Europe which has found that high severity of risk is predictive of poorer responses to universal interventions (8), suggests that high-risk youth may benefit from additional preventive education which is specifically tailored to their needs. In addition, we know that reasons for substance use are multifaceted and not only dependent on the social influence and normative use as is conveyed in most universal programs. Targeted programs offer the benefit of being able to focus on the role of other risk factors for addiction such as psychopathology and personality. However, such programs have often been overlooked due to their practical limitations as not only is it difficult to initially identify those individuals at greatest risk, but finding suitable, cost-effective ways to screen and deliver interventions can also be challenging (34). The targeted *Preventure* program overcomes these obstacles.

The targeted 'Preventure' program:

The school-based *Preventure* program is a brief personality-targeted substance use preventive intervention for high-risk adolescents aged 14-15 years. *Preventure* is the first and only targeted school-based alcohol and illicit drug prevention programme that has been shown to prevent growth in alcohol and substance misuse in Canada and the UK (5, 6, 45, 46). Unlike universal programs, this selected personality-targeted approach addresses four personality risk-factors for early-onset substance misuse and other risky behaviours: Sensation Seeking, Impulsivity, Anxiety Sensitivity and Hoplessness (47). The *Preventure* program is also consistent with new models which conceptualise substance use as being driven by personality traits such as impulsivity and disinhibition (48).The *Preventure* program involves two 90-minute group sessions which are carried out by a trained facilitator and co-facilitator.

The interventions are conducted using manuals which incorporate psycho-educational and cognitive behavioural components, and include real life scenarios shared by high-risk youth in specifically-organised focus groups. In the first session, participants are guided in a goal setting exercise, designed to enhance motivation to change behaviour. Psycho-educational strategies are used to teach participants about their target personality variable and associated problematic coping behaviours like avoidance, interpersonal dependence, aggression, risky behaviours and substance misuse. They are then introduced to the cognitive behavioural model and guided in breaking down personal experience according to the physical, cognitive and behavioural components of an emotional response. A novel component to this intervention approach is the fact that all exercises discuss thoughts, emotions and behaviours in a personality-specific way, e.g. identifying situational triggers and cognitive distortions related to Sensation Seeking specifically. In the second session, participants are encouraged to identify and challenge personality-specific cognitive distortions that lead to problematic behaviours.

The efficacy of the *Preventure* program has been demonstrated in a number of RCTs in Canada and the UK. Results from these trials revealed that *Preventure* successfully stemmed the growth in drinking and binge drinking in high-risk youth at six- and twelve-months

following the intervention (6), and more recent analysis has revealed the onset and escalation of drug misuse was prevented over a two-year period (5). In addition, *Preventure* has been shown to reduce emotional and behavioural problems specific to each of the personality profile (7). This is of particular importance given that comorbidity between substance use disorders and ill mental health is substantial and leads to worse outcomes (15).

Summary of the known and potential risks and benefits, if any, to human subjects

Based on prior evidence for the efficacy of the Climate Schools and Preventure programs (Castellanos & Conrod, 2006; Conrod et al., 2008; 2010; Newton et al., 2009a; 2009b), it is possible that students randomized to these groups will benefit from these programs. These programs have been shown to reduce along with associated harms including mental health co-morbidity.

At the start of each baseline and follow-up survey session the facilitator will make it clear to the students that their confidentiality will be strictly maintained. Students will also be made aware of their options if any of the issues raised in the survey has made them feel uncomfortable, sad or worried. The following will be explained to them (in addition to being included at the end of each questionnaire): ‘If any of the issues raised in this survey have made you feel uncomfortable, sad or worried, there are people you can reach out to. These could be your friends, family or teachers. You can also phone Kids Helpline on 1800 55 1800 for free, confidential help with any issues you want to talk about’.

It is possible that the *Preventure* sessions may uncover psychological distress in some participants. Systems will be in place to deal with such psychological distress. The Preventure sessions will be run by registered Psychologists who are trained to manage with individuals with current psychological distress. These Psychologists will also undergo specific training for implementing Preventure from CI Conrod, and this will involve how to deal with situations when students become distressed. Students will be reminded that they have the option of withdrawing from the study at any time without having an effect on their relationship with the school or the University of NSW. If they choose to withdraw due to psychological distress, the appropriate school staff member will be notified (with the consent of the student/parent?) and the necessary systems will be put in place.

Description of and justification for the route of administration, dosage, dosage regimen, and treatment period(s)

The *Climate Schools* program has been trialled and its efficacy as been established (Newton et al., 2009a; 2009b). The program consists of twelve 40-minute lessons and is designed to fit within the school health curriculum. It is facilitated by the internet which guarantees complete and consistent delivery whilst ensuring high implementation fidelity. It uses cartoon storylines to engage and maintain student interest and involvement over time (Schinke et al., 2004).

The *Preventure* program involves two 90-minute group sessions which are carried out by a trained facilitator and co-facilitator. The interventions are conducted using manuals which incorporate psycho-educational and cognitive behavioural components, and include real life scenarios shared by high-risk youth in specifically-organised focus groups. This program has also been trialled and its efficacy in this format has been established (Castellanos & Conrod, 2006; Conrod et al., 2008; 2010).

A statement that the trial will be conducted in compliance with the protocol, GCP and the applicable regulatory requirement(s)

This trial will be conducted in compliance with the protocol, the Guidelines for Good Clinical Practice, and the University of NSW Human Research Ethics Committee (HREC)

Description of the population to be studied

Participants will be recruited from schools within the greater Sydney region. They will be male and female and will be aged between 12-15 years at baseline. We aim to recruit 24 schools (approx 80 students per school), giving us a projected total of 1920 students at baseline.

References to literature and data that are relevant to the trial, and that provide background for the trial

1. Australian Institute of Health and Welfare. 2007 National Drug Strategy Household Survey: First results. Canberra: AIHW2008.
2. Newton N, Teesson M, Vogl L, Andrews G. Internet-based prevention for alcohol and cannabis use: final results of the Climate Schools course. Addictionin press;Accepted October 22nd 2009.
3. Newton NC, Andrews G, Teesson M, Vogl LE. Delivering prevention for alcohol and cannabis using the internet: A cluster randomised controlled trial. Preventive Medicine2009;48:579-84.
4. Newton NC, Vogl LE, Teesson M, Andrews G. CLIMATE Schools Alcohol Module: Cross-validation of a school-based prevention programme for alcohol misuse. Australian and New Zealand Journal of Psychiatry2009;43:201-7.
5. Conrod PJ, Castellanos N, Strang J. Brief, personality-targeted coping skills interventions prolong survival as a non-drug user over a two-year period during adolescence. Archives of General Psychiatry2010;67(1):85-93.
6. Conrod P, Castellanos N, Mackie C. Personality-targeted intervention delay the growth of adolescent drinking and binge drinking. Journal of Child Psychology and Psychiatry2008;49(2):181-90.
7. Castellanos N, Conrod P. Brief interventions targeting personality risk factors for adolescent substance misuse reduce depression, panic and risk taking behaviours. Journal of Mental health2006;15:645-58.

8. Faggiano F, Richardson C, Bohrn K, Galanti MR, Group EU-DS. A cluster randomized controlled trial of school-based prevention of tobacco, alcohol and drug use: The EU-Dap design and study population. Preventive Medicine2007 Feb;44(2):170-3.
9. Collins DJ, Lapsley HM. The costs of tobacco, alcohol and illicit drug abuse to Australian society in 2004/05. Canberra: Commonwealth of Australia 20082008.
10. Begg S, Vos T, Barker B, Stevenson C, Stanley L, Lopez AD. The burden of disease and injury in Australia 2003. Canberra: AIHW.2007.
11. Andrews G, Henderson S, Hall W. Prevalence, comorbidity, disability and service utilisation: Overview of the Australian National Mental Health Survey. British Journal of Psychiatry2001;178:145-53.
12. McLaren J, Mattick RP. Cannabis in Australia: Use, supply, harms, and response: Prepared by the National Drug and Alcohol Research Centre for the Drug Strategy Branch, Australian Government Department of Health and Ageing2007.
13. Copeland J, Swift W. Cannabis use disorder: epidemiology and management. International Review of Psychiatry2009;21:96103.
14. Loxley W, Toumbouruo JW, Stockwell T, Haines B, Scott K, Godfrey C, et al. The prevention of substance use, risk and harm in Australia: A review of the evidence. Canberra: Ministerial Council on Drug Strategy2004.
15. Teesson M, Degenhardt L, Hall W, Lynskey M, Toumbourou J, Patton G. Substance use and mental health in longitudinal perspective. In: Stockwall T, Grueneald P, Toumbourou J, Loxley W, editors. Preventing harmful substance use: The evidence base for policy and practice. Chichester: John Wiley and Sons; 2005.
16. Spooner C, Hall W. Public policy and the prevention of substance-use disorders. Current Opinion in Psychiatry2002;15(3):235-9.
17. Botvin GJ. Preventing drug abuse in schools: Social and competence enhancement approaches targeting individual-level etiologic factors. Addictive Behaviors2000;25(6):887-97.
18. Lloyd C, Joyce R, Hurry J, Ashton M. The effectiveness of primary school drug education. Drugs: Education, Prevention & Policy2000;7(2):109-26.
19. Cuijpers P. Effective ingredients of school-based drug prevention programs: A systematic review. Addictive Behaviors2002;27(6):1009-23.
20. Faggiano F, Vigna-Taglianti FD, Versino E, Zambon A, Borraccino A, Lemma P. School-based prevention for illicit drugs' use. Cochrane Database Syst Rev2005(2).
21. Hansen WB. School-based substance abuse prevention: A review of the state of the art in curriculum, 1980-1990. Health Education Research1992;7(3):403-30.
22. Tobler NS, Stratton HH. Effectiveness of school-based drug prevention programs: A meta-analysis of the research. Journal of Primary Prevention1997;18(1):71-128.
23. Tobler NS, Roona MR, Ochshorn P, Marshall DG, Streke AV, Stackpole KM. School-based adolescent drug prevention programs: 1998 meta-analysis. The Journal of Primary Prevention2000;20(4):275-336.
24. Gorman DM. Do school-based social skills training programs prevent alcohol use among young people? Addiction Research1996;4(2):191-210.
25. White D, Pitts M. Review: Educating young people about drugs: A systematic review. Addiction1998;93(10):1475-87.
26. Babor T, Caetano R, Casswell S, Edwards G, Giesbrecht N, Graham K, et al. Alcohol: No ordinary commodity. New York: Oxford Medical Publications; 2003.

27. Ennett ST, Rosenbaum DP, Flewelling RL, Bieler GS, et al. Long-term evaluation of Drug Abuse Resistance Education. Addictive Behaviors1994;19(2):113-25.
28. Werch CE, Moore MM, Diclemente CC, Owen DM, Carlson JM, Jobli E. Single vs. multiple drug prevention: Is more always better? A pilot study. Substance Use and Misuse2005;40:1085-101.
29. Munro G, Midford R. 'Zero tolerance' and drug education in Australian schools. Drug and Alcohol Review2001;20:105-9.
30. Beck J. 100 years of "just say no" versus "just say know": Re-evaluating drug education goals for the coming century. Evaluation Review1998;22(1):15-45.
31. Botvin GJ. Advancing prevention science and practice: Challenges, critical issues, and furture directions. Prevention Science2004;5(1):69-72.
32. Ennett ST, Ringwalt CL, Thorne J, Rohrbach LA, Vincus A, Simons-Rudolph A, et al. A comparison of current practice in school-based substance use prevention programs with meta-analysis findings. Prevention Science2003;4(1 Mar):1-14.
33. Bosworth K. Application of computer technology to drug abuse prevention. In: Sloboda Z, Bukoski WJ, editors. Handbook of Drug Abuse Prevention: Theory, Science and Practice. New York: Kluwer Academic/ Plenum Publishers; 2003. p. 629-48.
34. Offord DR. Selection of levels of prevention. Addictive Behaviours2000;25(6):833-42.
35. Mrazek PJ, Haggerty RJ. Reducing risks for mental disorders: Frontiers for prevention intervention research. Washington DC: National Academy Press; 1994.
36. Jones L, Sumnall H, Burrell K, McVeigh J, Bellis MA. Universal drug prevention. Liverpool, UK: National Collaborating Centre for Drug Prevention2006.
37. Midford R. Is this the path to effective prevention? Addiction2008;103(7):1169-70.
38. Botvin GJ, Griffin KW. School-based programmes to prevent alcohol, tobacco and other drug use. International Review of Psychiatry2007 Dec;19(6):607-15.
39. Cuijpers P. Three decades of drug prevention research. Drugs: education, prevention and policy2003;10(1):6-20.
40. Morgenstern M, Wiborg G, Isensee B, Hanewinkel R. School-based alcohol education: Results of a cluster-randomized controlled trial. Addiction2009;104:402-12.
41. McBride N, Farringdon F, Muleners L, Midford R. School Health and Alcohol Harm Reduction Project: Details of intervention development and research procedures. Perth, W.A.: National Drug Research Institute, Curtin University of Technology2006.
42. Vogl L, Teesson M, Andrews G, Bird K, Steadman B, Dillon P. A computerised harm minimisation prevention program for alcohol misuse and related harms: Randomised controlled trial. Addiction2009;104:564-75.
43. Schinke S, Schwinn TM, Noia JD, Cole KC. Reducing the risks of alcohol use among urban youth: Three-year effects of a computer-based intervention with and without parent involvement. Journal of Studies on Alcohol2004;65:443-9.
44. Vogl L, Newton N, Teesson M, Swift W, Karageorge A, Deans C, et al. Climate Schools: Drug prevention programs. Sydney: National Drug and Alcohol Research Centre, UNSW2009.
45. Castellanos N, Conrod P. Personality and substance misuse: Evidence for a four factor model of vulnerability. In: Verster J, Brady K, Strain E, Galanter M, Conrod PJ, editors. Drug Abuse and Addiction in Medical Illness: Humana/Spring Press; in press.
46. Conrod P, Stewart SH, Comeau N, Maclean AM. Preventative efficacy of cognitive behavioural strategies matched to the motivational bases of alcohol misuse in at-risk youth. Journal of Clinical Child Adolescent Psychology2006;35:55-563.

47. Woicik PB, Conrod P, Stewart SH, Pihl RO. The Substance Use Risk Profile Scale: A scale measuring traits linked to reinforcement-specific substance use profiles. Addictive Behaviours2009;32:1042-55.
48. Dick DM, Aliev F, Wang JC, Grucza RA, Schuckit M, Kuperman S, et al. Using dimensional models of externalizing psychopathology to aid in gene identification. Archives of General Psychiatry2008;65:310–8.
49. Heo M, Leon AC. Sample size requirements to detect an intervention by time interaction in longitudinal cluster randomized clinical trials. Statistics in Medicine2009;28:1017-27.
50. McBride N, Farringdon F, Midford R, Meuleners L, Phillips M. Harm minimisation in school drug education: Final results of the School Health and Alcohol Harm Reducation Project (SHAHRP). Addiction2004;99:278-91.
51. Martin G, Copeland J, Gilmour S, Gates P, Swift W. The adolescent cannabis problems questionnaire (CPQ-A): Psychometric properties. Addictive behaviours2006;31(12):2238-48
52. Kovacs M. *CDI: Children's Depression Inventory: Technical manual update*. Toronto: Multi Health Systems2005.
53. Marsch LA, Bickel WK, Badger GJ. Applying computer technology to substance abuse prevention science: Results of a preliminary examination. Journal of Child & Adolescent Substance Abuse2007;16(2):69-94.
54. Clark DB, Winters KM. Measuring risks and outcomes in substance use disorders prevention research. Journal of Consulting & Clinical Psychology2002;70:1207-23.
55. Crowley TJ, Mikulich SK, Ehlers KM, Whitmore AE, Macdonald MJ. Validity of structured clinical evaluations in adolescents with conduct and substance problems. Journal of American Acadamy for Child and Adolescent Psychiatry2001;40:256-73.
56. Carrol KM. Methodological issues and problems in the assessment of substance use. Psychological Assessment1995;7(3):349-58.
57. Raudenbush SW, Bryk AS, Congdon RT. HLM 6: Hierarchical Linear Modelling and Non Linear Modelling. Lincolnwood, IL: Scientific Softwarre International, Inc.; 2004.
58. Lee VE. Using hierarchical linear modelling to study social contexts: The case of school effects. Educational Psychologist2000;35(2):125-41.

Trial Objectives and Purpose

Detailed description of the objectives and the purpose of the trial

Overall Aim:
To evaluate the first comprehensive 'universal' and 'targeted' model to prevent substance use and related harms in Australian adolescents.

The aim of the current proposal is to combine the effective universal *Climate* and targeted *Preventure* programs into a comprehensive model to prevent substance use and related harms in adolescents. This model will be known as the *CAP intervention* and will result in a sequential approach to drug prevention which overcomes traditional implementation obstacles to school-based prevention and is consistent with new models that conceptualise substance use as existing on a continuum (49). Delivering prevention using the proposed comprehensive approach offers a way of preventing substance use at a whole population level and has the potential to maximize outcomes for both high- and low-risk youth. This study will be the first time such intervention has been trailed, and will also be the first trial of a targeted substance use prevention program in Australia.

Hypotheses related to trial outcomes:
The comprehensive school-based *CAP intervention*, which combines effective 'universal' and 'targeted' approaches to substance use prevention, will be more effective than;
(1) treatment as usual, (2) stand-alone 'universal' drug prevention, and (3) stand-alone 'targeted' drug prevention, in:

- a) reducing the uptake and harmful use of alcohol and illicit substances
- b) reducing substance use related harms, and
- c) reducing rates of mental health co-morbitity.

In addition, for high-risk students only, the evidence-based targeted *Preventure* program will be more effective than; (1) treatment as usual, and (2) stand-alone 'universal' drug prevention, in:

- a) reducing the uptake and harmful use of alcohol and illicit substances
- b) reducing substance use related harms, and
- c) reducing rates of mental health co-morbitity.

Trial Design

A specific statement of the primary endpoints and the secondary endpoints, if any, to be measured during the trial

The implementation of Climate Schools and Preventure programs will finish by the end of Term 3 (21st September) in 2012 and the final assessment surveys will be administered by the end of Term 1 (10th April) in 2015.

A description of the type/design of trial to be conducted (e.g. double-blind, placebo-controlled, parallel design) and a schematic diagram of trial design, procedures and stages

This study will employ a clustered randomized controlled trial. Cluster randomization was chosen to avoid contamination of the control group. Schools will be randomly allocated to one of four groups (6 schools per group); (1) the 'Control' condition (usual classes), (2) the 'Climate Schools only' condition, (3) the '*Preventure* only' condition, or (4) the '*CAP (Climate Schools and Preventure)*' condition. The flow chart of allocation of schools to intervention groups is depicted in Figure 1.

Figure 1. Flow chart of allocation of schools to the intervention groups

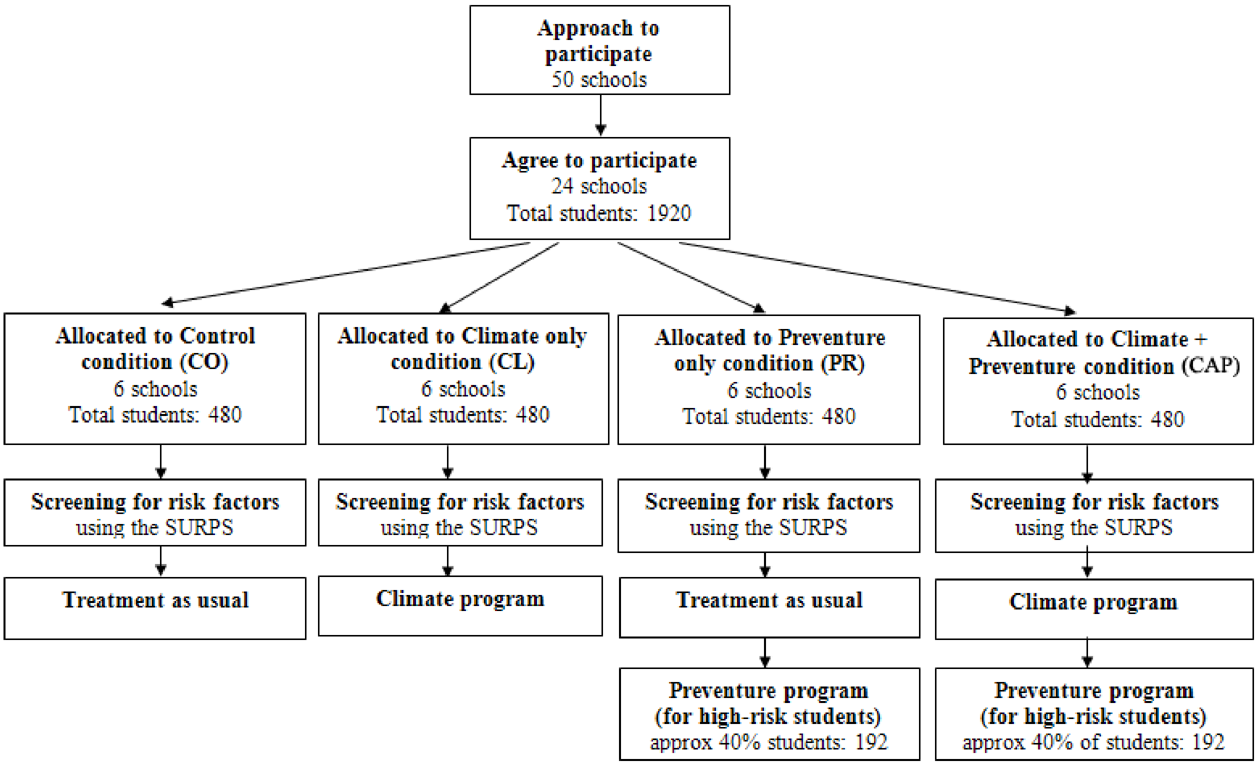

Table 1 illustrates the stages of intervention and assessment. Baseline assessments and four follow-up assessments will be carried out over the study period. All parent consent forms will go out in the first 2-3 weeks of Term 1 (6th Feb - 17th Feb) and all Baselines (with student consent) will be implemented as soon as possible after this date (aim to finish those on March 9th- gives 3 weeks). Therefore 6 month surveys will be done in September before the end of Term 3 (with room for some to do it in Term 4).

As soon as they complete the baselines they can start the Climate intervention. Preventure will be run in Terms 2 and 3 (with a couple of schools run in Term 1 when CI Conrod comes to Australia to train the clinicians).

Table 1: CAP study assessment and intervention timeline

	Baseline Survey (including SURPS)	*Climate* program	*Preventure* program (high-risk students)	6 month F/U Survey	12 month F/U Survey	24 month F/U Survey	36 month F/U Survey
Time	Term 1 Feb-March 2012	Terms 1 and 3 Feb-Sept 2012	Terms 2 - 3 April-Sept 2012	Term 3 Sept 2012	Term 1 Feb-March 2013	Term 1 Feb-March 2014	Term 1 Feb-March 2015
Grade	Year 8	Year 8	Year 8	Year 8	Year 9	Year 10	Year 11
**CO***	✓			✓	✓	✓	✓
**CL***	✓	✓		✓	✓	✓	✓
**PR***	✓		✓	✓	✓	✓	✓
**CAP***	✓	✓	✓	✓	✓	✓	✓

*CO: Control group, CL: CLIMATE only group, PR: PREVENTURE only group, and CAP: CLIMATE plus PREVENTURE group.

A description of the measures taken to minimize/avoid bias, including: (a) Randomization. (b) Blinding.

In order the minimize/avoid bias, schools will be randomised to the study groups. Balanced randomisation will occur using the online program Research Randomizer. In addition, the Research Officers employed to carry out the baseline and follow-up assessments will be blinded to the group allocations of the schools.

A description of the trial treatment(s) and the dosage and dosage regimen of the investigational product(s). Also include a description of the dosage form, packaging, and labelling of the investigational product(s)

The Climate Schools program:

The Climate Schools program aims to reduce the use of Australia's most commonly used licit and illicit drugs: alcohol and cannabis, and related harms. The program was pioneered by CI Teesson, CI Newton and AI Andrews and is based on the effective harm-minimisation approach to prevention (Newton et al., 2009a; 2009b; 2009c; McBride et al., 2006; Vogl et al., 2009). The Climate Schools program consists of twelve 40-minute lessons and is designed to fit within the school health curriculum. It is facilitated by the internet which guarantees complete and consistent delivery whilst ensuring high implementation fidelity. It uses cartoon storylines to engage and maintain student interest and involvement over time (Schinke et al., 2004). The first six lessons focus specifically on alcohol and are delivered approximately six month prior to the remaining six lessons which focus on cannabis. The first part of each lesson is completed individually over the internet where students are engaged through cartoon storylines which impart information about the short- and long-term effects of alcohol and cannabis, normative alcohol and cannabis use, refusal skills, and first aid. Students are provided with confidential login details to access the Climate website. The second part of each lesson is a group or class activity delivered by the teacher which reinforces the information in the cartoons and allows interactive communication between students. Teachers are provided with a manual containing the activities, implementation guidelines, links to the education syllabus and summaries for each lesson.(To view the Climate Schools program, visit www.climateschools.com and logon using the Username: 'Teacher' and Password: 'London').

The Preventure program:

The Preventure program is a brief personality-targeted substance use preventive intervention for high-risk adolescents aged 14-15 years. Unlike universal programs, this selected personality-targeted approach addresses four personality risk-factors for early-onset substance misuse and other risky behaviours: Sensation Seeking, Impulsivity, Anxiety Sensitivity and Hoplessness (Woicik et al., 2009). The Preventure program is also consistent with new models which conceptualise substance use as being driven by personality traits such as impulsivity and disinhibition (Dick et al., 2008).This program involves two 90-minute group sessions which are carried out by a trained facilitator and co-facilitator. subIn the first session, participants are guided in a goal setting exercise, designed to enhance motivation to change behaviour. Psycho-educational strategies are used to teach participants about their target personality variable and associated problematic coping behaviours like avoidance, interpersonal dependence, aggression, risky behaviours and substance misuse. They are then introduced to the cognitive behavioural model and guided in breaking down personal experience according to the physical, cognitive and behavioural components of an emotional response. A novel component to this intervention approach is the fact that all exercises discuss thoughts, emotions and behaviours in a personality-specific way, e.g. identifying situational triggers and cognitive distortions related to Sensation Seeking specifically. In the second session, participants are encouraged to identify and challenge personality-specific cognitive distortions that lead to problematic behaviours. Preventure is the first and only targeted school-based alcohol and illicit drug prevention programme that

has been shown to prevent growth in alcohol and substance misuse in Canada and the UK (Conrod et al., 2006; 2008; 2010).

The CAP Intervention:
The CAP intervention will combine the Climate Schools and Preventure programs with the aim of providing a comprehensive 'universal' and 'targeted' model to prevent substance use and related harms in Australian adolescents. The CAP study has logos that will be used on all study materials (see Figure 2).

Figure 2. The CAP study logos

The expected duration of subject participation, and a description of the sequence and duration of all trial periods, including follow-up, if any.

See Table 1 for the intervention and assessment timeline. Participants will be recruited at the start of Year 8 and baselines will be carried out in Term 1. The Climate Schools and Preventure programs will be implemented in Terms 1-3. Follow up assessments will occur during Years 8, 9, 10 and the start of Year 11. Hence participants will in the study for approximately 3 years.

A description of the "stopping rules" or "discontinuation criteria" for individual subjects, parts of trial and entire trial

Is it possible that some participants may decide to discontinue with the Preventure program due to emotional distress. Students and will be informed and reminded throughout the study that participation is voluntary they have the option of withdrawing from the study at any time. It will be emphasised that withdrawal from the study will have no effect on their relationship with the school or the University of NSW. If they choose to discontinue they will be referred to the appropriate school staff member/counsellor (with their consent).

Accountability procedures for the investigational product(s), including the placebo(s) and comparator(s), if any

N/A

Maintenance of trial treatment randomization codes and procedures for breaking codes

Balanced randomization of schools to conditions will be implemented using the online program Research Randomizer (http://www.randomizer.org/).

The identification of any data to be recorded directly on the Case Report Forms (i.e. no prior written or electronic record of data), and to be considered to be source data

N/A

Selection and Withdrawal of Subjects

Subject inclusion criteria

Participants are male and female students in Year 8 who are willing to consent to participating in the study. Parental/guardian consent will also be obtained. Participants invited to take part in the Preventure program will be screened as 'high risk' using the Substance Use Risk Profile Scale (SURPS).

Subject exclusion criteria

There are no exclusion criteria for this study.

Subject withdrawal criteria

(i.e. terminating investigational product treatment/trial treatment) and procedures specifying:
(a) When and how to withdraw subjects from the trial/ investigational product treatment.
(b) The type and timing of the data to be collected for withdrawn subjects.
(c) Whether and how subjects are to be replaced.
(d) The follow-up for subjects withdrawn from investigational product treatment/trial treatment.

There are no withdrawal criteria for this study. However, students and will be informed and reminded throughout the study that participation is voluntary they have the option of withdrawing from the study at any time. If a student decides to withdraw, replacements will not be sought.

Treatment of Subjects

The treatment(s) to be administered, including the name(s) of all the product(s), the dose(s), the dosing schedule(s), the route/mode(s) of administration, and the treatment period(s), including the follow-up period(s) for subjects for each investigational product treatment/trial treatment group/arm of the trial.

Table 1 illustrates the intervention and assessment periods for the trial. All participants will be asked to complete the baseline and follow-up surveys. Schools will be randomised to one of four groups; (1) the 'Control' condition (usual classes/assessment only), (2) the 'Climate Schools only' condition, (3) the '*Preventure* only' condition, or (4) the '*CAP (Climate Schools and Preventure)*' condition.

The Climate Schools program:
The Climate Schools program aims to reduce the use of Australia's most commonly used licit and illicit drugs: alcohol and cannabis, and related harms. The program was pioneered by CI Teesson, CI Newton and AI Andrews and is based on the effective harm-minimisation approach to prevention (Newton et al., 2009a; 2009b; 2009c; McBride et al., 2006; Vogl et al., 2009). The Climate Schools program consists of twelve 40-minute lessons and is designed to fit within the school health curriculum. It is facilitated by the internet which guarantees complete and consistent delivery whilst ensuring high implementation fidelity. It uses cartoon storylines to engage and maintain student interest and involvement over time (Schinke et al., 2004). The first six lessons focus specifically on alcohol and are delivered approximately six month prior to the remaining six lessons which focus on cannabis. The first part of each lesson is completed individually over the internet where students are engaged through cartoon storylines which impart information about the short- and long-term effects of alcohol and cannabis, normative alcohol and cannabis use, refusal skills, and first aid. Students are provided with confidential login details to access the Climate website. The second part of each lesson is a group or class activity delivered by the teacher which reinforces the information in the cartoons and allows interactive communication between students. Teachers are provided with a manual containing the activities, implementation guidelines, links to the education syllabus and summaries for each lesson.(To view the Climate Schools program, visit www.climateschools.com and logon using the Username: 'Teacher' and Password: 'London').

The Preventure program:
The Preventure program is a brief personality-targeted substance use preventive intervention for high-risk adolescents aged 14-15 years. Unlike universal programs, this selected personality-targeted approach addresses four personality risk-factors for early-onset substance misuse and other risky behaviours: Sensation Seeking, Impulsivity, Anxiety Sensitivity and Hoplessness (Woicik et al., 2009). The Preventure program is also consistent with new models which conceptualise substance use as being driven by personality traits such as impulsivity and disinhibition (Dick et al., 2008).This program involves two 90-minute group sessions which are carried out by a trained facilitator and co-facilitator. subIn the first session, participants are guided in a goal setting exercise, designed to enhance motivation to change behaviour. Psycho-educational strategies are used to teach participants about their target personality variable and associated problematic coping behaviours like

avoidance, interpersonal dependence, aggression, risky behaviours and substance misuse. They are then introduced to the cognitive behavioural model and guided in breaking down personal experience according to the physical, cognitive and behavioural components of an emotional response. A novel component to this intervention approach is the fact that all exercises discuss thoughts, emotions and behaviours in a personality-specific way, e.g. identifying situational triggers and cognitive distortions related to Sensation Seeking specifically. In the second session, participants are encouraged to identify and challenge personality-specific cognitive distortions that lead to problematic behaviours. Preventure is the first and only targeted school-based alcohol and illicit drug prevention programme that has been shown to prevent growth in alcohol and substance misuse in Canada and the UK (Conrod et al., 2006; 2008; 2010).

The CAP Intervention:
The CAP intervention will combine the Climate Schools and Preventure programs with the aim of providing a comprehensive ‘universal’ and ‘targeted’ model to prevent substance use and related harms in Australian adolescents. The CAP study has a logo that will be used on all study materials (see Figure 1).

Medication(s)/treatment(s) permitted (including rescue medication) and not permitted before and/or during the trial

N/A

Procedures for monitoring subject compliance

Participants in the Climate Schools condition will be required to attend sessions as they will be implemented as part of the PDHPE school classes. Those in the Preventure condition will be sent reminder emails for the two focus groups. The number of sessions attended will be recorded for each participant.

Assessment of Efficacy

Specification of the efficacy parameters

Treatment efficacy will be determined by analysing changes in three outcome measures over the study period:

1. The uptake and harmful use of alcohol and illicit substances
2. Substance use related harms
3. Mental health comorbidity

This information will be gleaned from the baseline and follow-up assessments using reliable and well-validated measures.

The assessment surveys include the following measures:

1. *Demographic data* including gender, age, academic performance, and truancy rates will be obtained to determine baseline equivalence of the groups
2. *The Substance Use Risk Profile Scale (SURPS)* will be used to assess for variation in personality risk for substance abuse/dependence along 4 dimensions: Sensation Seeking, Impulsivity, Anxiety Sensitivity and Hopelessness. Students who score more than one SD above the school mean on any of the four personality risk subscales will be categorized into these sub-groups and will be invited to participate in the Prevention intervention if they are randomised into the CAP or Preventure groups. If a student has high scores on more than one subscale, they will be assigned to the group in which they showed the most statistical deviance according to z-scores;
3. *Alcohol and cannabis knowledge, Patterns of Alcohol use, Cannabis use and Other substance use,* and *Attitudes about alcohol* will be assessed using questionnaires adapted from the School Health and Alcohol Harm Reduction Project (SHAHRP; McBride et al., 2003; 2006) and also based on the questions from the National Drug Strategy Household Survey 2007;
4. *Harms caused by cannabis use* will be measured using 12 items from the SHAHRP survey instrument and a set of questions adapted from the Adolescent Cannabis Problems Questionnaire (Martin et al., 2006);
5. Five questions will be used to assess student's *intention to use alcohol and illicit drugs* in the 'future'
6. *The Resistive Self-Regulatory Efficacy Scale* will be used to measure participants' perceived efficacy to resist peer pressure to engage in high risk activities (Bandura et al., 2003);
7. The *Kessler 6* scale (Kessler et al., 2002) will be used to assess past-month psychological distress
8. The *Strengths and Difficulties Questionnaire* (Goodman, 1998) will be used to assess both positive and negative attributes of the students;
9. *Depression and Anxiety symptoms* will be measured using the depression and anxiety subscales from the Brief Symptom Inventory (BSI; Derogatis 1993);
10. The *Reactive-Proactive Aggression Questionnaire* (Raine et al., 2006) will be used to measure students' levels of reactive and proactive aggression;
11. The *Bullying Questionnaire* comprises questions taken from a large international study entitled Health Behaviour in School-aged Children (HBSC), and are originally

taken from the revised Olweus Bully/Victim Questionnaire (Olweus, 1996), and the Youth Risky Behaviour Survey (Brener et al., 1995);

12. The *Thoughts and Feelings Questionnaire* is a brief questionnaire on psychotic symptoms derived from the Personal Assessment and Crisis Evaluation (PACE, Phillips et al., 2000) and the Prodromal Questionnaire (PQ; Loewy et al., 2005);
13. Symptoms of alcohol abuse will be assessed using the Diagnostic Interview Schedule for Children (DISC-IV; Shaffer et al., 2000).

Methods and timing for assessing, recording, and analysing of efficacy parameters

Assessment surveys will be administered at baseline, and at 6, 12, 18, 24, 30, and 36 months post-baseline. Participating schools have the option of completing these assessments online or by paper-and-pencil. Data will be collated and analysed at each assessment stage using PASW Statistics 18. Hierarchical Linear Modelling (HLM) will be used for clustered data.

Assessment of Safety

Specification of safety parameters

It is possible that the Preventure sessions may uncover psychological distress in some participants. These sessions will be run by registered Psychologists who are trained to manage individuals with current psychological distress. These Psychologists will undergo specific training for implementing Preventure and this will involve how to deal with situations where students become distressed.

The methods and timing for assessing, recording, and analysing safety parameters

The Psychologists running the Preventure sessions will be trained to identify and deal with situations in which participants become distressed.

Procedures for eliciting reports of and for recording and reporting adverse event and intercurrent illnesses

Situations in which participants in the Preventure sessions become distressed will be recorded in the Psychologists session notes. The Psychologists will also be required to report these incidents to the project co-ordinator and will then decide if any further action is necessary and whether the appropriate school staff member will need to be notified (with the consent of the student).

The type and duration of the follow-up of subjects after adverse events

If students find the Preventure sessions too distressing they will be reminded that they have the option of withdrawing from the study at any time. If the Psychologists and project manager deem it necessary (and with the consent of the student) an appropriate school staff member will be notified. The student will then be in the care of the staff member and will be dealt with according to school policy.

Statistics

A description of the statistical methods to be employed, including timing of any planned interim analysis(ses)

Baseline equivalence and attrition between groups will be examined using single-level analyses; one-way analyses of variance to examine normally distributed data, Chi-square to examine binominal data, and Mann-Whitney U-test to examine non-normally distributed data. To examine intervention by time interaction effects, mixed effects regression will be used due to the multi-level and hierarchical nature of the data. To account for intracluster correlations between schools, intervention effects will primarily be examined using hierarchical linear modeling (HLM) for normally distributed data and hierarchical generalized linear modeling using Poisson sampling for count data. Outcome variables will be centered at post-test to allow for comparisons between groups immediately after the intervention and growth terms will be analysed to determine the magnitude of the follow-up effects. Analyses will be conducted using the program HLM 6 (57). If unconditional models reveal that less than 10% of systematic variance exists at the between-school level for any outcome variable, HLM will be abandoned and single-level analyses will be used (58). For these variables, ANCOVAs utilising the SPSS GLM procedure will be conducted to account for any baseline differences that might exist between groups. Bonferroni adjustments will be made for multiple comparisons. Odds ratios and 95% confidence intervals will also be calculated.

Interim analyses will take place at each assessment stage (including the baseline assessment). This will assist with the monitoring of data collection and data entry. Final stage analyses will be carried out at the 36 month follow-up.

The number of subjects planned to be enrolled

In multicentre trials, the numbers of enrolled subjects projected for each trial site should be specified. Reason for choice of sample size, including reflections on (or calculations of) the power of the trial and clinical justification

To account for cluster randomisation, sample size calculations are based on recent sample size requirements developed by Heo & Leon (2009) to detect intervention by time interactions in longitudinal cluster randomized clinical trials. This trial will be powered to detect differences in the overall sample as well as in the high-risk students also. To allow for comparisons between the high-risk students, 600 high risk students from 20 schools are required (i.e. 30 high-risk students per school and 5 schools per intervention group). This would achieve 80% power to detect a standardized between-group mean difference of 0.3 ($p = 0.05$) in outcomes at the end of the trial with 7 measurement occasions. An effect size of 0.3 is comparable to previous trials of drug prevention programs (Cuijpers, 2002; Tobler & Stratton, 1997). To account for dropouts during the trial which we expect to be approximately 10% (Newton et al., 2009a; Conrod et al., 2010), we aim to recruit 80 students per school, 40% ($n = 32$) of whom are expected to be high-risk based on previous research CI Conrod, and 6 schools per group giving us a total of 1920 students from 24 schools at baseline to test the effect of the intervention in the overall group.

The level of significance to be used

All tests conducted will use a predetermined alpha level of .05.

Criteria for the termination of the trial

There are no criteria for the termination of the trial. There have been no significant adverse events in prior research to indicate termination is necessary.

Procedure for accounting for missing, unused, and spurious data

The appropriate longitudinal statistical methods will be used that account for missing data.

Procedures for reporting any deviation(s) from the original statistical plan

(any deviation(s) from the original statistical plan should be described and justified in protocol and/or in the final report, as appropriate)

Any deviations from the original statistical plan will be described and justified in an amendment to this protocol.

The selection of subjects to be included in the analyses

(e.g. all randomized subjects, all dosed subjects, all eligible subjects, evaluable subjects)

All randomised participants will be included in the overall efficacy analyses.

Direct Access to Source Data/Documents

The sponsor should ensure that it is specified in the protocol or other written agreement that the investigator(s)/institution(s) will permit trial-related monitoring, audits, IRB/IEC review, and regulatory inspection(s), providing direct access to source data/documents.

In relation to data and safety monitoring, the Chief Investigaors and the Project Coordinator will meet regularly with the Research Officers and Research Psychologists to assure the proper conduct of the study and the safety of participants. This will involve monitoring the data collection and data entry and reviewing any adverse event reports that may arise.

Progress reports will be submitted annually to the NHMRC by 31st March (for the previous year). This report will provide information relevant to the achievements of aims of the study for each of the 5 years of funding.

Figure 3. NHMRC Progress Report template

PROGRESS REPORT ON RESEARCH ACTIVITY

The National Health and Medical Research Council (NHMRC) requires all NHMRC Administering Institutions that have signed a Funding Agreement with NHMRC to continue to meet the requirements of the Funding Agreement. The Funding Agreement requires the Administering Institution to ensure that a Progress Report prepared by the Specified Person in relation to an ongoing Research Activity is submitted to NHMRC by the date specified in the Funding Policy.

The purpose of the Progress Report is to collect information relevant to the achievements of aims against those specified in the application for each Research Activity.

This form should be used for reporting on the following types of funding schemes:

- **CRE grants**
- **Development Grants**
- **Partnership project Grants**
- **Program Grants**
- **Project Grants (including European Union Projects)**
- **Strategic Awards (including Targeted Calls for Research and NHMRC EU Collaborative Research Grants)**

Information provided in the Progress Report will be used to assist NHMRC to satisfy legislative requirements in relation to its administration of funding. Reports will be assessed by NHMRC staff. Failure to complete and submit this report may result in funding of a Research Activity being suspended until the Report is provided.

A signed electronic version of the Progress Report should be emailed by your RAO to: postaward.management@nhmrc.gov.au.

Questions regarding Progress Reports should be referred to the Research Administration Section by telephoning 1800 500 983 or emailing postaward.management@nhmrc.gov.au

Figure 3 (continued). NHMRC Progress Report template

SECTION A – Administration details

**Administering Institution**	
**NHMRC Application ID**	
**Chief Investigator A**	
**Title of Research Project**	
**Type of Research Grant**<br>**(eg. Project Grant)**	
**Year of Funding to which this report applies (eg. 1,2,3 etc)**	

SECTION B –Progress against the research aims

1. Have the aims or objectives outlined in the application changed since funding was awarded for the project?
   (Please circle a response) YES NO
2. If the answer to Q1 is YES, please explain why the aims have changed (eg. If aims changed because the amount of funding sought was reduced, please advise below)

Figure 3 (continued). NHMRC Progress Report template

3. Is satisfactory progress towards achieving the stated research aims being achieved?
   (Please circle a response) YES NO

4. If the answer to Q3 is NO, please explain why satisfactory progress has not been achieved

5. Has your on-line CV been updated to reflect all current publications associated with this research activity?
   (Please circle a response) YES NO

(Note: In examining reports, NHMRC may wish to use details of publications contained within the CV loaded in RGMS. Therefore it is preferable that this is kept up to date.)

SECTION C – Declaration

This section should be signed by the CIA and the responsible officer or their delegate.

I declare that:

- This report accurately reflects the status of the funded project and that I understand that I am required to provide accurate information to NHMRC; and
- Relevant Institutional Approvals have been maintained to date in accordance with the relevant NHMRC Funding Agreement.

Signature of Chief Investigator A<br><br>…………………………………………………………………………	Date<br><br>………/………/………
Signature of Responsible Officer or delegate<br><br>…………………………………………………………………………	Date<br><br>………/………/………

Quality Control and Quality Assurance

The Climate Schools intervention and assessments will be administered online so that quality is controlled and assured.

Ethics

Please see the attached UNSW HREC Ethics Application (HREC 11274) and associated attachments. Also refer to the UNSW HREC reply (dated 22/07/2011) and reply from the Investigators (dated 3/8/2011).

Data Handling and Record Keeping

All computer files containing the collected data will be password protected and only those directly involved in the project will have access. Hard copy consent forms, student lists (including student’s unique codes to link their surveys over time), and de-identified surveys, will be stored separately in locked filing cabinets. Only the research staff directly involved in the project will have access to them. Data collected as part of the assessment and treatment components of the trial will be recorded in PASW Statistics 18 by the Research Officers employed on the study.

Financing and Insurance

TYPE OF FUNDING
NHMRC Project Grant - Standard Project Grant

PERIOD OF FUNDING
5 year(s) of funding has been approved for this project
Start date 1 January 2011
End date 31 December 2015
Final date by which the project must commence 30 June 2011

FUNDS, FUNDING - Total Funding amount is: $723665

APPROVED BUDGET

**Year**	1	2	3	4	5
**TOTAL**	$178902	$224599	$143205	$143205	$33754

Funding Payments are scheduled quarterly in advance.
Note: GST and an annual indexation may be applied to some components of this budget.

**This information was obtained from NHMRC App 1004744 Schedule.*

This project will be carried out in accordance with UNSW insurance policies.

Publication Policy

Findings will be published in peer reviewed journals throughout the trial and at the conclusion. All CIs and research staff on the time will be involved in preparing these for submission. Participating schools will also be sent the results and have the option to share these with parents and students.

UNSW Human Research Ethics Application Form

More than Low Risk OR Low Risk Research Applications

Project Description Template

1. Lay Summary & Aims (Maximum of 500 words)
Briefly provide a summary of the project in lay terminology (no more than 500 words).<br>The project summary should include the following details:<br>• Aims of the project<br>• Importance of the study

In 2011, CIs Newton, Teesson, Slade and Conrod (NHMRC APP1004744; HREC 11274) initiated the *Climate and Preventure (*CAP) study: the first ever randomised controlled trial (RCT) of a comprehensive school-based drug prevention model combining two complementary approaches: *universal* prevention, which is delivered to all irrespective of level of risk, and *selective* prevention, which is tailored for adolescents with personality vulnerabilities that increase risk for alcohol and other drug use. Twenty-six schools and 2,190 participants (aged 13-14) were recruited to the CAP study in 2012 and students completed five self-report assessments over 3 years. Participant retention is high, ranging from 71 to 83% over the 3-year follow up. Results at 3 years demonstrate the effectiveness of universal *and* selective approaches in preventing harmful alcohol use among low- and high-risk adolescents, the additive benefits of universal and selective prevention for reducing cannabis uptake in high-risk adolescents, and the effectiveness of both universal and selective prevention in reducing aggression among high-risk adolescents. Worldwide, little is known about the effectiveness of drug prevention approaches beyond 3 years. Therefore, the aim of the present study is to extend the follow-up of the CAP Study cohort over the critical transition from adolescence into early adulthood.

Original ethics approval (HREC 11274) expired on the 15th of August 2016 and a 3-month extension was subsequently granted. We are now seeking approval to conduct ongoing research to determine the long-term effectiveness of the CAP intervention. In particular, this research aims to answer the following questions:

1. What is the long-term effectiveness of universal, selective and combined alcohol and drug prevention into early adulthood (ages 18-21)?
2. Are selective and universal alcohol and drug prevention effective in reducing aggressive behaviour and violent offending into early adulthood?
3. What are the mechanisms of change underlying the intervention effects?

This project will be the first in the world to examine whether combining *universal* and *selective* drug prevention strategies enhances durability of effects in the longer-term, over a 7-year period from adolescence to early adulthood.
This is an important transitional period with unique challenges and increased susceptibility to alcohol and cannabis use and related harms. It will answer critical questions about the potential benefits of these approaches in reducing alcohol and drug-related violence during the high-risk early adulthood period. Further, it will provide crucial information about the underlying mechanisms of change and inform future refinements to achieve even more powerful prevention effects.

2. Background Literature Review – (Maximum of 500 words)
Provide an outline of the theoretical background for the research proposal with reference to relevant literature.<br>• The background literature review should be "based on a thorough study of the current literature, as well as previous studies" (NS 1.1c) along with;<br>• The potential significance of the study

Young adulthood in Australia is characterised by increased use of alcohol and cannabis (Teesson et al., 2010; Hall et al., 2016). Within a 12 month period, 1 in 3 young Australian adults consume alcohol at very high risk levels and 1 in 5 use cannabis (AIHW, 2013). At this age, episodes of intoxication can have significant health, legal, social and financial consequences, including risk of impaired neurocognitive function and development (Hall et al., 2016). In addition, patterns of use established during early adulthood increase risk of developing substance use disorders and co-morbid mental health problems, all of which negatively impact on current functioning and future life options (Hall et al., 2016). Among young people aged 20-24, alcohol and other drug use is among the leading contributors to total burden of death, disease and injury, accounting for an estimated 14% of total disease burden (Degenhardt et al., 2016). The substantial harm related to drug and alcohol use is in part attributable to the associated risk of violent behaviour, including assaults, homicides, self-harm and suicide (Hall et al., 2016). For example, toxicology reports from a national coronial inquiry in Australia found that alcohol and/or drug (primarily cannabis) use was involved in 78% of "King hit" (single incapacitating blow to the head) cases between 2000 and 2012 (Pilgrim et al., 2014).

Despite the considerable harms attributable to alcohol and drug use, our prevention evidence base in this age group is

weak. Adolescents with personality risk factors for alcohol and other drug use, such as impulsiveness and sensation-seeking (Castellanos & Conrod, 2012; Newton et al., 2016), may be particularly susceptible during this period of new found autonomy. The transition to young adulthood also encompasses legal access to alcohol at 18 years in Australia, and increasingly regular drinking or other drug use may emerge as a way of forming new friendships and/or coping with the increasing life stressors and demands that emerge at this stage. Our own investigations highlight the importance of coping motives for alcohol use (i.e. drinking to cope with negative emotions such as anxiety or hopelessness), which are strongly associated with alcohol-related harms during this early period of adulthood (Stapinski et al., 2016). Despite the unique challenges and increased susceptibility during this transitional period, few studies have examined the effectiveness of drug and alcohol prevention approaches beyond secondary school. There is limited evidence from studies in the United States (Spoth et al., 2016; Spoth et al., 2015; Ellickson et al., 1993) that receiving the universal Botvin Life Skills Training in Year 6-7 (ages 12-13) reduced risk of alcohol-related problems and illicit drug use into early adulthood (ages 18-22). The long-term effectiveness of selective prevention approaches (such as *Preventure*), and the combination of skills development within a social influence model (such as *Climate*) is unknown.

The CAP trial cohort is now approaching early adulthood and represents a unique opportunity to examine the impact of universal and selective prevention on developmental pathways to alcohol and cannabis harms in young adults. These two prevention approaches have different strengths: while universal approaches have the capacity to reduce alcohol and drug use on a wider scale (Botvin et al., 2007), selective approaches are optimal for youth at high risk of drug and alcohol misuse due to an underlying vulnerability (Conrod et al., 2008). Along with increased exposure to alcohol and cannabis during early adulthood, high-risk personality styles may become more pronounced with the onset of new challenges, increased autonomy, and formation of new friendship circles (Elkins et al., 2006). The CAP study provides a unique opportunity to examine whether high-risk young people are less responsive to universal interventions (Faggiano et al., 2007), and may gain greater benefits in the longer-term from selective programs which target the underlying risk factors for drug and alcohol use (Conrod et al., 2008).

The durability of universal and selective prevention effects into early adulthood is a crucial research question, as the choices young people make during this important life stage can have profound effects for many years to come. The CAP trial is the first in Australia to adapt and evaluate the selective *Preventure* program, and the first ever trial of a comprehensive prevention model combining selective and universal interventions. Our international team is uniquely placed to capitalise on this window of opportunity with our existing trial cohort to address the gap in knowledge about longer-term outcomes of alcohol and drug prevention.

3. Research Design and Methodology (Maximum 1 page)

Provide an outline of the research design, the study timeline and data collection methods.

Study Design

Participating schools in the CAP study (2012-2015) were randomised to one of four conditions: Climate, Preventure, Climate and Preventure (CAP) or control (see *Figure 2*). Following the baseline assessment at the start of Term 1, students in the intervention groups received the (online) *Climate Schools* and/or (2 session, manualised) *Preventure* programs during Terms 1 and 3 of Year 8.

The ***Climate Schools*** program is based on the effective harm-minimisation approach to prevention and aims to reduce the use of alcohol, cannabis and related harms among young people through computer-delivered cartoon storylines which impart information about the short- and long-term effects of alcohol and cannabis, normative alcohol and cannabis use, refusal skills, and first aid. The program was pioneered by CI Teesson, CI Newton and AI Andrews (Newton et al., 2009a; 2009b; 2009c; McBride et al., 2006; Vogl et al., 2009) and consists of twelve 40-minute lessons and is designed to fit within the school health curriculum.

The ***Preventure*** program is a brief personality-targeted substance use preventive intervention for high-risk adolescents aged 14-15 years. Young people with high scores on four personality risk-factors for early-onset substance misuse and other risky behaviours (i.e. Sensation Seeking, Impulsivity, Anxiety Sensitivity and Hoplessness (Woicik et al., 2009)) participate in two 90 minute group sessions with a trained facilitator and co-facilitator aimed at addressing maladaptive coping behaviours. Only students who scored 'high-risk' on any one of the four personality subscales on the SURPS received the *Preventure* only program.

Students in the CAP condition received a combined intervention involving the universal Climate Schools intervention as well as the targeted Preventure intervention. Students randomised to the control group received their usual health education

classes (including lessons on drugs and alcohol), and were offered the use of the *CAP intervention* following completion of the study.

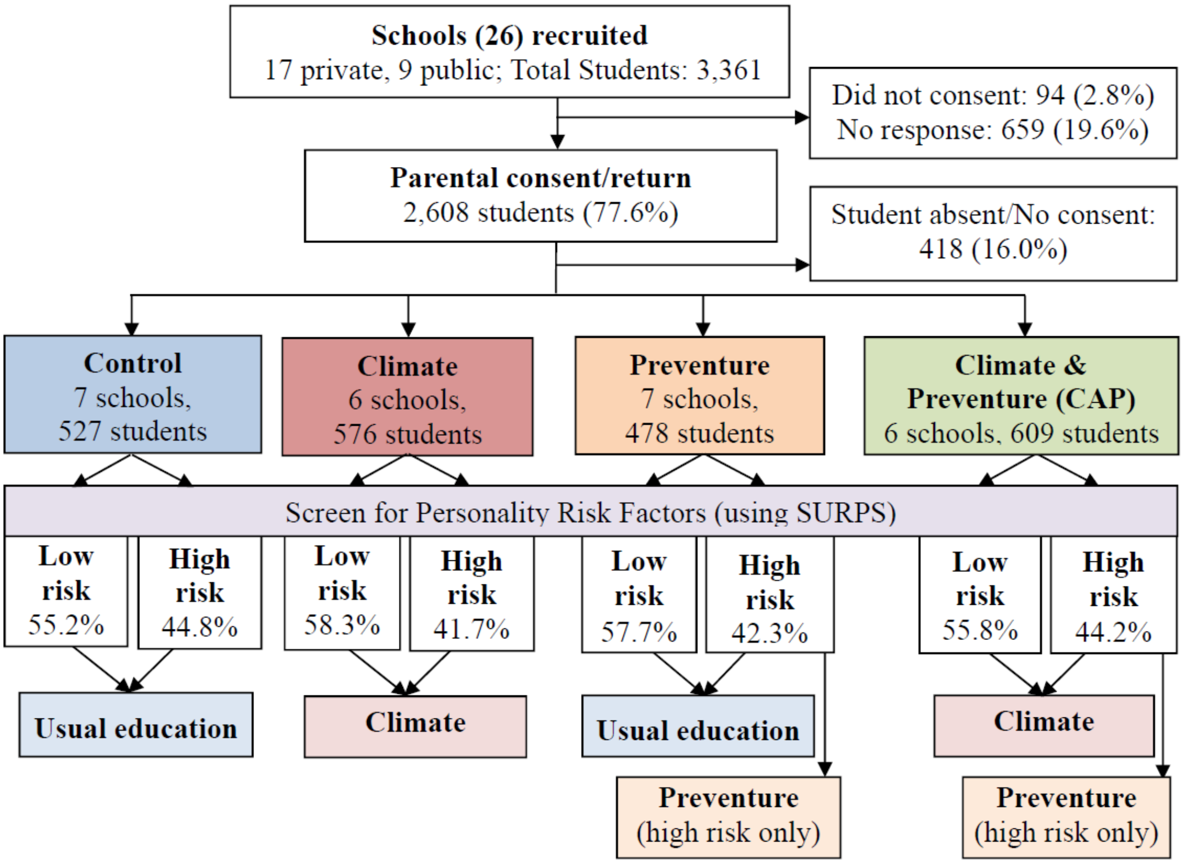

Figure 2. CONSORT diagram showing design and participants recruited into the original CAP trial.

Schedule of Assessments
Table 1 shows assessments competed as part of the original CAP trial, and proposed extended follow-up assessments. Using multiple sources of locator information already provided to the research team, all participants will be invited to complete online assessments at 5-, 6- and 7- years. Participants will complete surveys online via the CAP study website (www.capstudy.org.au). Participant responses will be linked over time using a unique identification code to ensure confidentiality.

Table 1: Completed CAP study assessments and timeline for extended follow-up assessments

**Original CAP trial**				**Timeline for Extended Follow-up**		
2012	2013	2014	2015	2017	2018	2019
Year 8	Year 9	Year 10	Year 11	Post-school +1	Post-school +2	Post-school +3
Baseline + Intervention n = 2,190	1-year F/U survey n = 1,818 (83%)	2-year F/U survey n =1,732 (79%)	3-year F/U survey n =1,601 (71%)	5-year F/U survey ⟷	6-year F/U survey ⟷	7-year F/U ⟷
Court Records of Drug and Violent Offending accessed through retrospective data linkage ⟷						
Note. Results will be disseminated via conference presentations, peer-reviewed journal articles, community webinars and teacher workshops.				Statistical Analyses ⟷		
					Dissemination of results ⟷	

Measures
Demographic data including gender, age, academic performance, and truancy rates have been collected at baseline. To allow for modelling of outcomes over a 7-year period, extended follow-up measures will be consistent with those administered during the original trial. All assessments will involve self-report using well validated instruments. Self-report is the favoured method of assessment for young people for assessment of drug and alcohol use symptoms and has been found to have excellent discriminant and predictive validity (Crowley et al., 2001). To corroborate reports of aggression and alcohol/drug related harms, consent will be sought to match participant data with court record data held in the Reoffending Database at the NSW Bureau of Crime Statistics and Research (BOCSAR).

a) Primary and Secondary Outcomes (RQ1 and RQ2)
Binge-drinking and Cannabis use will be assessed using an adapted version of the Patterns of Alcohol index (McBride et al., 2004), and cannabis questions from the National Drug Strategy Household Survey 2013 (AIHW, 2013). These measures allow for comparison with a large scale representative group of Australians. *Alcohol and Cannabis-related harms* will be measured using an abbreviated version of the Rutgers Alcohol Problem Index (White et al., 1989); and a 6-item scale will be used to assess cannabis related harms (Copeland et al., 2005). The DSM-5 self-report symptom checklist developed by Batterham et al (2016) will be administered to assess emerging symptoms of alcohol and other drug use disorders and other psychiatric symptoms as participants enter adulthood. *Aggression* will be measured using the Reactive-Proactive Aggression Questionnaire (Raine et al., 2006). Self-reported violence will be measured using a 7 item violence scale validated in the Dunedin longitudinal study (Arseneault et al., 2000). To corroborate self-report measures and to further examine the criminal involvement of this group as well as hypothesised intervention effects on drug-related violent offending, consent will be sought to match participant data with court record data from the Bureau of Crime Statistics and Research (BOSCAR). The Reoffending Database contains detailed court records of every individual who has appeared in the Children's, Local, District and Supreme Criminal Courts of NSW since 1994. Drug-related offences over the period from 2012 (baseline) to 2019 and violent offending (homicide, assault) from 2012 to 2019 will be examined through data linkage to the Reoffending Database. To analyse mortality outcomes and to ensure that no deceased persons are contacted to participate in follow-up surveys consent will also be sought to link CAP study participant survey data with the National Death Index at the Australian Institute of Health and Welfare.

b) Hypothesised Mechanisms of Change (RQ3)
Drinking motives across four dimensions (social, conformity, enhancement, and coping motives) will be assessed by the Drinking Motives Questionnaire-Revised (Grant et al., 2009). High risk personality traits will be assessed using the *Substance Use Risk Profile Scale (SURPS)* personality questionnaire (Woicik et al., 2009). *History of trauma* will be examined using the Life Events Checklist (Weathers et al., 2013a). *Perceived peer use of alcohol and cannabis* will be measured using 5 items (Newton et al., 2010) that gauge participants' estimation of the proportion of their friends who consume alcohol and cannabis. *Self-efficacy to resist peer influences* will be assessed using an adapted 8-item version of Bandura's Resistive Self-Regulatory Efficacy Scale (Newton et al., 2014; Bandura et al., 2003). *Emotional and behavioural problems* will be assessed using the Kessler-6. Specific depression and anxiety symptoms will be measured by relevant subscales of the Brief Symptom Inventory (Derogatis et al., 1993). Capacity to modulate emotional reactions and associated behaviours will be assessed with the Emotion Regulation Questionnaire (Gross et al., 2003). Sleep, diet and sedentary behaviour will be measured using items from the International Physical Activity Questionnaires as adapted by Ding et al. (2015).

4. Sample Size (Maximum of 250 Words)

Outline the intended sample size for the project and justify how this will meet the aims of the study.

Sample size calculations tools can be found online at:
- http://powerandsamplesize.com/Calculators/ (quantitative)
- http://stat.ubc.ca/~rollin/stats/ssize/index.html (quantitative)
- http://eprints.ncrm.ac.uk/2273/4/how_many_interviews.pdf (qualitative)

Participants for this study are 2,190 individuals who were recruited to the original CAP study as students in 2012. Power calculations for the original trial were based on recent methods developed to account for cluster randomisation (Heo et al., 2009). These calculations assumed 20% drop-out, and indicated a total of 1,920 participants recruited from 24 schools would achieve 80% power to detect a between-group mean difference of 0.15 ($p < 0.05$). For comparisons among high risk participants, 600 students from 20 schools would achieve 80% power to detect a standardized between-group mean difference of 0.3. In our original study we achieved well over this recruitment target, with 2,190 students and 947 high-risk students participating in the study.

5. Research Participants (Maximum of 500 words)

Describe the characteristics of the participants that you intend to recruit in the study (e.g. inclusion/exclusion criteria, sex, age range of participants).

Participants for this study are 2,190 young people (57% male) from 26 Sydney schools recruited to the original CAP study in 2012 who have provided their non-school locator information online. At the 5 year follow up in 2017 almost all participants will be between 18 and 19 years of age. Study participants who have actively withdrawn consent to participate will not be contacted. All remaining subjects will be contacted to and invited to consent to participate in Survey 5, 6 and 7.

6. Recruitment of participants

Outline the methods that will be used to recruit participants to the study. The methods in this section should describe:

- What process(es) will be used to identify potential participants;
- How initial contact will be made with potential participants;
- How participants will be screened to ensure they meet the inclusion criteria.

Participants will be located using multiple sources of locator information already provided by the subject. This includes email, phone number, postal address, Facebook handle, and parents' email address. Participants will be invited to complete online assessments at the 5 (2017), 6 (2018) and 7 (2019) year time points. Participants will be contacted in 2017 and invited to participate in the extended follow-up study. Only participants who consent will be subsequently contacted by project staff. Study participants who have actively withdrawn consent to participate will not be contacted.

7. Consent

Provide a detailed description of the consent process to include the following:

- The type of consent that will be sought (e.g. verbal/ written/ return of survey etc.), how and when you will provide consent materials to your potential participants and why this method of consent is appropriate for the participant population.
- How, when and to whom participants will indicate their consent and how any real or perceived coercion will be avoided during the consent process.

**if the project involves the use of data already collected and the participants have already provided their consent for this to happen, please attach a copy of the original consent form as evidence.
**if you are seeking a waiver of consent, please provide justification. (See the National Statement, Chapters 2.2 and 2.3 for more information).

In line with follow-up procedures used in waves 1-4, participants will be contacted via email or phone in 2017 and will be invited to consent to completing three additional surveys. Participants will also be asked for consent for their name and date of birth to be released to the Reoffending Database at the Bureau of Crime Statistics and Research (BOCSAR), and to the National Death Index (NDI) Database at the Australian Institute of Health and Welfare (AIHW).

Linking to the Reoffending Database at BOCSAR will allow for an objective measure of drug and alcohol related problems and self-reported aggression and criminal involvement. This is particularly important given the strong association between drug use and crime where two in every three offenders (66%) who are detained by police are under the influence of least one drug – not including alcohol (Payne & Gaffney, 2012). Participants will be advised that, should consent be given for data linkage, the information collected will be offence type, date of offence and penalty. Participants will be asked to consent to data linkage occurring on three occasions (2017, 2018 & 2019). Participants will be informed that this information will be collected to better understand the relationship between drug use and crime with the aim of informing prevention initiatives.

Linking of participant data to the NDI at the AIHW will allow for a deeper understanding of the relationship between substance use, mental health, psychological and environmental factors. Participants will be informed of the exact information that will be obtained from the NDI. Participants will be advised that, should consent be given, the information that will requested will be all information stored in the NDI database, fact of death information (e.g. date of death, state/territory the death was registered in, year the death was registered) and cause of death information (e.g. underlying and other causes of death). Participants will also be asked for consent to repeat data linkage with NDI i.e. for each of the three follow-up survey occasions in 2017, 2018 and 2019. Participants will be informed that this data will be utilised to analyse survival rates and mortality outcomes of CAP study participants, and also to ensure that no deceased persons are contacted to participate in follow-up surveys.

It will be stressed to participants that they are not required to consent to the data linkage, and can with withdraw consent from data linkage or other aspects of the study at any time. The participant information sheets state clearly that involvement in the study is voluntary and that a decision whether or not to participate will not prejudice future relations with The University of New South Wales or the researchers (see attachment). It also states that participants are free to

withdraw at any time without giving a reason and without penalty.

8. Reimbursement of Expenses or Incentives to Participate

Explain whether there will be any reimbursement of out-of-pocket expenses, financial incentive or other "reward" as a result of participation in the study.

All participants will go into the draw to win an iPad (valued at $400) or a $400 JB HIFI voucher at the end of each survey occasion. In addition, those who complete all surveys (5, 6 & 7) will go in a draw to win an iPad at the end of the follow-up period (one per 2 schools). Participants will not be reimbursed for consenting to the data linkage component of the study.

9. Risks to participants

Describe the anticipated risks to the participants, including:

- Whether the benefits outweigh the risks to the participants;
- How will you manage or minimise the risks to participants.

There are no major foreseeable risks involved with participation in this study. Although unlikely, it is possible that some individuals may experience emotional discomfort as the questionnaire asks about use of alcohol and other drugs and mental health symptoms. It is also possible that participants may feel some mild distress as this questionnaire may ask you to disclose information about engaging in illegal activity (i.e. consuming illicit drugs). Participants will be made aware that they have the right to stop completing the questionnaire at any time, and that they can withdraw from the study. Participants will also be provided with contact details of the study co-ordinators, for support services (e.g. Lifeline) and the Alcohol and Drug Information Service at the end of the survey.

BOSCAR Reoffending Database (ROD) linkage – Potential Risks: There are no major risks associated with providing consent to record linkage with the ROD as confidentiality of this information will be strictly maintained by BOSCAR and the study investigators. Only de-identified information will be used for the purposes of analysis and any research findings will be published in a way that ensures that participating individuals cannot be identified.

AIHW National Death Index (NDI) database linkage – Potential Risks: There are no major risks associated with providing consent to record linkage with the NDI. All information will remain strictly confidential and will be maintained by the Registrars of Births, Deaths and Marriages, AIHW and the study investigators. Only de-identified information will be used for the purposes of analysis and any research findings will be published in a way that ensures that participating individuals cannot be identified.

10. Privacy and Confidentiality

Outline the methods that will be applied to ensure that the privacy of participants is protected during:

- The recording and analyses of data;
- The storage of data;
- Publishing or reporting the information.

Confidentiality of collected information will be strictly maintained and all data collected will be done anonymously so there will be no identifying information on any data collected. The survey information collected from the participants will be allocated a username (for online surveys). Participants who do not have access to the internet and require a paper and pencil survey will be given a special code. This is for the purposes of linking the survey data collected at each follow up stage. At no stage will the participants' names be connected with their data i.e. not on the original data collected or in the saved data files.

Online data: To access the online surveys, participants will be required to logon to the CAP study website using their unique username (email address) and password. Once logged in, all data collected will automatically be de-identified and the database will generate a unique code for each participant. The individual's data files across sessions will be linked with this unique code.

SSL (Secure Sockets Layer) Security will be used to ensure any confidential communication via the website (e.g. participant registration and online surveys) is kept secure. SSL provides a mechanism to verify the identity of an internet client and/or server, and to encrypt the messages sent between them. All data will be stored on secure serves hosted in Australia.

Paper data: Some participants may wish to complete surveys with pen and paper rather than online. In this case, participants use a unique code (made up of easily remembered fragments of personal information) each time they fill in a survey and this code will be used to link their surveys over time. Only the researcher will have access to these codes.

No information will be published on individual cases, with all published data being group data.

BOCSAR Reoffending Data: In order for data linkage to occur, identifiable information would need to be released to BOCSAR (so long as participant consent is provided for this data to be shared). Confidentiality of this data would be strictly maintained by BOCSAR. BOCSAR has minimized the risk to personal privacy by ensuring that the data will be stored on a password protected computer network in a secure location. BOCSAR has a Code of Practice under Part 3 Division 1 of the Privacy and Personal Information Protection Act 1998. The Code of Practice was developed in consultation with the Privacy Commissioner and made by order of the Attorney General. This code of practice has provisions to ensure that BOCSAR does not disclose personal information provided by agencies.

AIHW National Death Index database: Identifiable information would be required to be released to the AIHW in order for record linkage to occur. There is an agreement between the AIHW and the Registrars of Births, Deaths and Marriages, which covers the restrictions on the use and release of the information provided. Under this agreement, the Registrars are regularly notified of all NDI linkage projects. A Registrar is a member of the AIHW Ethics Committee, and the confidentiality conditions of the AIHW Act 1987 apply to data provided by NDI users.

11. Publications and Dissemination of Results

In this section detail how:

- The research results will be reported to the participants of the study;
- How the research results will be reported/ published;
- How participant confidentiality will be maintained in your reports and/or publications.

Results will be published in peer reviewed academic journals and disseminated via conference presentations, community webinars and teacher workshops. Participating schools and individuals will not be identified at any point in the publication and dissemination of the study findings. Feedback will be provided through a de-identified report of the study's findings that will be sent to participating schools and made available to participants.

References

1. Bandura, A., Caprara, G. V., Barbaranelli, C., Gerbino, M., & Pastorelli, C. (2003). Role of affective self-regulatory efficacy in diverse spheres of psychosocial functioning. *Child Development*, 74, 769–782.
2. Teesson, M., Hall, W., Slade, T., Mills, K., Grove, R., Mewton, L., Ballie, A. & Haber, P. (2010) Prevalence and correlates of DSM-IV alcohol abuse and dependence in Australia: findings of the 2007 NSMHWB. *Addiction*. 105: 2085-94.
3. Hall, W., Patton, G., Stockings, E., Weier, M., Lynskey, M., Morley, K., & Degenhardt, L. (2016). Why young people's substance use matters for global health. *The Lancet Psychiatry*, *3*(3), 265-279.
4. Australian Institute of Health and Welfare (2014). National Drug Strategy Household Survey detailed report 2013. Drug statistics series no. 28. Cat. no. PHE 183. Canberra: AIHW.
5. Degenhardt, L., Stockings, E., Patton, G., Hall, W. D., & Lynskey, M. (2016). The increasing global health priority of substance use in young people. *The Lancet Psychiatry*, *3*(3), 251-264.
6. Pilgrim, J. L., Gerostamoulos, D., & Drummer, O. H. (2014). "King hit" fatalities in Australia, 2000–2012: The role of alcohol and other drugs. *Drug and alcohol dependence*, *135*, 119-132.
7. Botvin, G. J., & Griffin, K. W. (2007). School-based programmes to prevent alcohol, tobacco and other drug use. *International review of psychiatry*, *19*(6), 607-615.
8. Conrod, P. J., Castellanos, N., & Mackie, C. (2008). Personality-targeted interventions delay the growth of adolescent drinking and binge drinking. *Journal of Child Psychology and Psychiatry*, *49*(2), 181-190.
9. Elkins, I. J., King, S. M., McGue, M., & Iacono, W. G. (2006). Personality traits and the development of nicotine, alcohol, and illicit drug disorders: prospective links from adolescence to young adulthood. *Journal of abnormal psychology*, *115*(1), 26.
10. Faggiano, F., Richardson, C., Bohrn, K., Galanti, M. R., & EU-Dap Study Group. (2007). A cluster randomized controlled trial of school-based prevention of tobacco, alcohol and drug use: The EU-Dap design and study population. *Preventive medicine*, *44*(2), 170-173.

11. Newton, N. C., Vogl, L. E., Teesson, M., & Andrews, G. (2009). CLIMATE Schools: alcohol module: cross-validation of a school-based prevention programme for alcohol misuse. *Australian and New Zealand Journal of Psychiatry*, *43*(3), 201-207.
12. Newton, N. C., Teesson, M., Vogl, L. E., & Andrews, G. (2010). Internet-based prevention for alcohol and cannabis use: final results of the Climate Schools course. *Addiction*, *105*(4), 749-759.
13. Castellanos-Ryan, N., & Conrod, P. (2012). Personality and substance misuse: evidence for a four-factor model of vulnerability. In *Drug Abuse and Addiction in Medical Illness* (pp. 47-62). Springer New York.
14. Newton, N. C., Conrod, P., Slade, T., Carragher, N., Champion, K., Barrett, E., Kelly, E., Nair, N., Stapinski, L. & Teesson, M. (2016). The long-term effectiveness of a selective, personality-targeted prevention program in reducing alcohol use and related harms: a cluster randomized controlled trial. *Journal of child psychology and psychiatry*.
15. Stapinski, L. A., Edwards, A. C., Hickman, M., Araya, R., Teesson, M., Newton, N. C., Kendler, K. & Heron, J. (2016). Drinking to cope: a latent class analysis of coping motives for alcohol use in a large cohort of adolescents. *Prevention Science*, *17*(5), 584-594.
16. Spoth, R. L., Clair, S., Shin, C., & Redmond, C. (2006). Long-term effects of universal preventive interventions on methamphetamine use among adolescents. *Archives of pediatrics & adolescent medicine*, *160*(9), 876-882.
17. Spoth, R., Trudeau, L., Redmond, C., & Shin, C. (2014). Replication RCT of early universal prevention effects on young adult substance misuse. *Journal of consulting and clinical psychology*, *82*(6), 949.
18. Ellickson, P. L., Bell, R. M., & McGuigan, K. (1993). Preventing adolescent drug use: long-term results of a junior high program. *American Journal of Public Health*, *83*(6), 856-861.
19. Newton, N. C., Barrett, E. L., Swaffield, L., & Teesson, M. (2014). Risky cognitions associated with adolescent alcohol misuse: moral disengagement, alcohol expectancies and perceived self-regulatory efficacy. *Addictive behaviors*, *39*(1), 165-172.
20. Crowley, T. J., Mikulich, S. K., Ehlers, K. M., Whitmore, E. A., & Macdonald, M. J. (2001). Validity of structured clinical evaluations in adolescents with conduct and substance problems. *Journal of the American Academy of Child & Adolescent Psychiatry*, *40*(3), 265-273.
21. McBride, N., Farringdon, F., Midford, R., Meuleners, L., & Phillips, M. (2004). Harm minimization in school drug education: final results of the School Health and Alcohol Harm Reduction Project (SHAHRP). *Addiction*, *99*(3), 278-291.
22. Copeland, J., Gilmour, S., Gates, P., & Swift, W. (2005). The Cannabis Problems Questionnaire: factor structure, reliability, and validity. *Drug and Alcohol Dependence*, *80*(3), 313-319.
23. Raine, A., Dodge, K., Loeber, R., Gatzke-Kopp, L., Lynam, D., Reynolds, C., ... & Liu, J. (2006). The reactive–proactive aggression questionnaire: Differential correlates of reactive and proactive aggression in adolescent boys. *Aggressive behavior*, *32*(2), 159-171.
24. Grant, V., Stewart, S. & Mohr, C. (2009). Evaluation of the Drinking Motives Questionnaire. *Addict Behav*. 32(2): 226-237.
25. Derogatis L. (1993). Administration, scoring and procedures manual for the brief symptom inventory (4th ed.). Minneapolis, MN: National Computer Systems, 1993.
26. Gross, J. J., & John, O. P. (2003). Individual differences in two emotion regulation processes: implications for affect, relationships, and well-being. *Journal of personality and social psychology*, *85*(2), 348.
27. Batterham, P. J., Sunderland, M., Carragher, N., Calear, A. L., Mackinnon, A. J., & Slade, T. (2016). The Distress Questionnaire-5: Population screener for psychological distress was more accurate than the K6/K10. *Journal of clinical epidemiology*, *71*, 35-42.
28. Ding, D., Grunseit, A. C., Chau, J. Y., Vo, K., Byles, J., & Bauman, A. E. (2016). Retirement—A Transition to a Healthier Lifestyle?: Evidence From a Large Australian Study. *American journal of preventive medicine*, in press.
