# Supplementary material for: Effect of Selective Personality-Targeted Alcohol Use Prevention on 7-Year Alcohol-Related Outcomes Among High-risk Adolescents: A Secondary Analysis of a Cluster Randomized Clinical Trial
Source: JAMA Netw Open. 2022 Nov 17;5(11):e2242544. doi: 10.1001/jamanetworkopen.2022.42544 (PMC9672969; doi:10.1001/jamanetworkopen.2022.42544)
Supplement: Supplement 2. — eMethods. Intervention Description eAppendix 1. Graphical Representations of Results eFigure 1. Monthly Binge Drinking in Past 6 Months (≥5 Standard Drinks on 1 Occasion) eFigure 2. Any Alcohol-Related Harms in the Past 6 Months eFigure 3. Cumulative Alcohol-Related Harms eFigure 4. AUDIT-C Hazardous Drinking Scores (>3) eAppendix 2. Attrition Analysis eTable 1. Baseline Differences Between Those Lost to Follow-up and Those Present at the Long-term Follow-up eAppendix 3. Sensitivity Analyses eTable 2. Relative Change in Log Odds of Alcohol Outcomes at 7 Years Post Baseline (Adjusted for Baseline Differences in Sex) eTable 3. Relative Change in Odds and Mean Frequency of Alcohol Outcomes and Mean Frequency of Alcohol Harms From Baseline to 5.5 Years [file jamanetwopen-e2242544-s002.pdf]

Supplementary Online Content

Newton NC, Debenham J, Slade T, et al. Effect of selective personality-targeted alcohol use prevention on 7-year alcohol-related outcomes among high-risk adolescents: a secondary analysis of a cluster randomized clinical trial. *JAMA Netw Open*. 2022;5(11):e2242544. doi:10.1001/jamanetworkopen.2022.42544

eMethods. Intervention Description
eAppendix 1. Graphical Representations of Results
eAppendix 2. Attrition Analysis
eAppendix 3. Sensitivity Analyses

This supplementary material has been provided by the authors to give readers additional information about their work.

This supplemental material has been provided by the authors to give readers additional information about their work.

© 2022 Newton NC et al. *JAMA Network Open*.

eMethods. Intervention Description

Preventure is a brief personality-targeted, selective intervention comprising two 90-minute sessions delivered one week apart. Sessions are conducted separately for, and tailored to, each personality group. For example, all participants who scored at least one standard deviation above the mean on the Sensation Seeking subscale, formed one group and received a version of *Preventure* tailored to the Sensation Seeking risk profile. Students with elevated scores on more than one SURPS subscale (n=132, 30% of baseline sample) were allocated to the personality group corresponding to that of their subscale score which deviated most from the mean, according to z scores. Sessions were delivered by trained facilitators in adherence to the training protocol described by O'Leary-Barrett et al. (2010)[34]. Facilitators were registered clinical psychologists and co-facilitators were research assistants with Bachelor of Psychology (Honours) qualifications.

The two sessions incorporate psycho-education, motivation enhacement therapy and cognitive behavioural therapy components into real life situations to explore helpful and unhelpful coping strategies common to each personality profile and goal-setting exercises. Students are introduced to the cognitive-behavioural model through examination of their emotional, behavioural and physiological reactions to a recent experience, and then are supported to identify and challenge profile-specific cognitions that precede problematic behaviours.

© 2022 Newton NC et al. *JAMA Network Open*.

eAppendix 1. Graphical Representations of Results

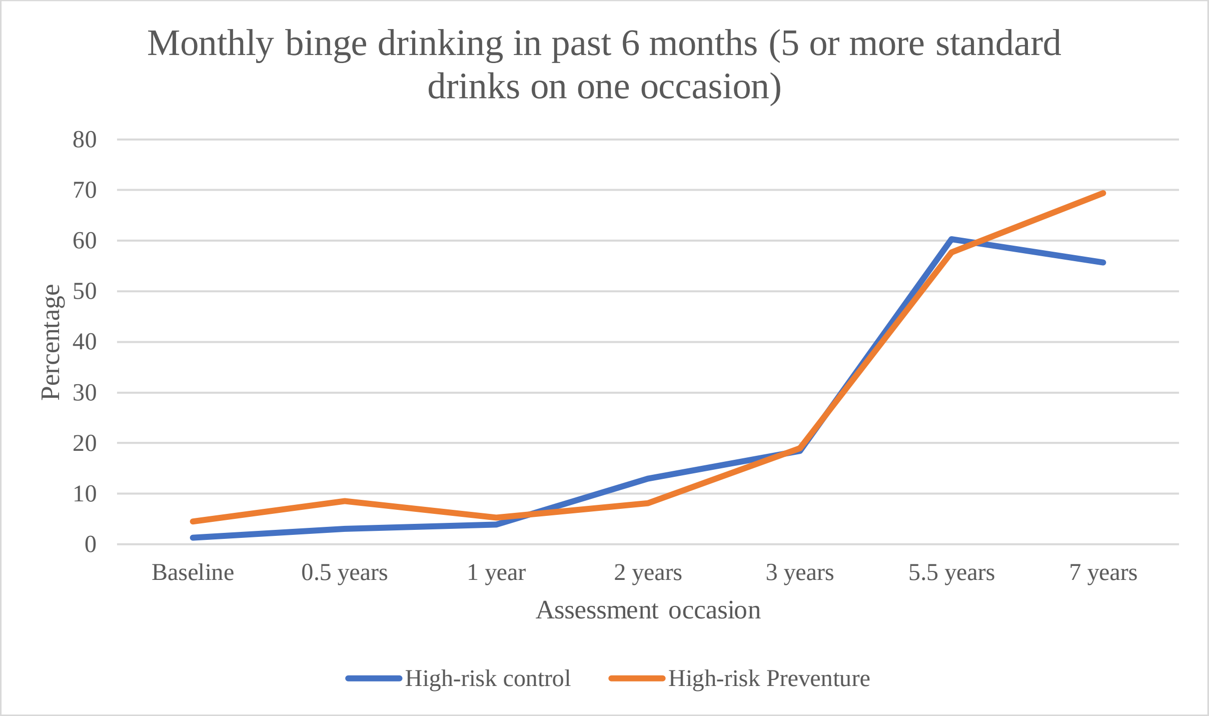

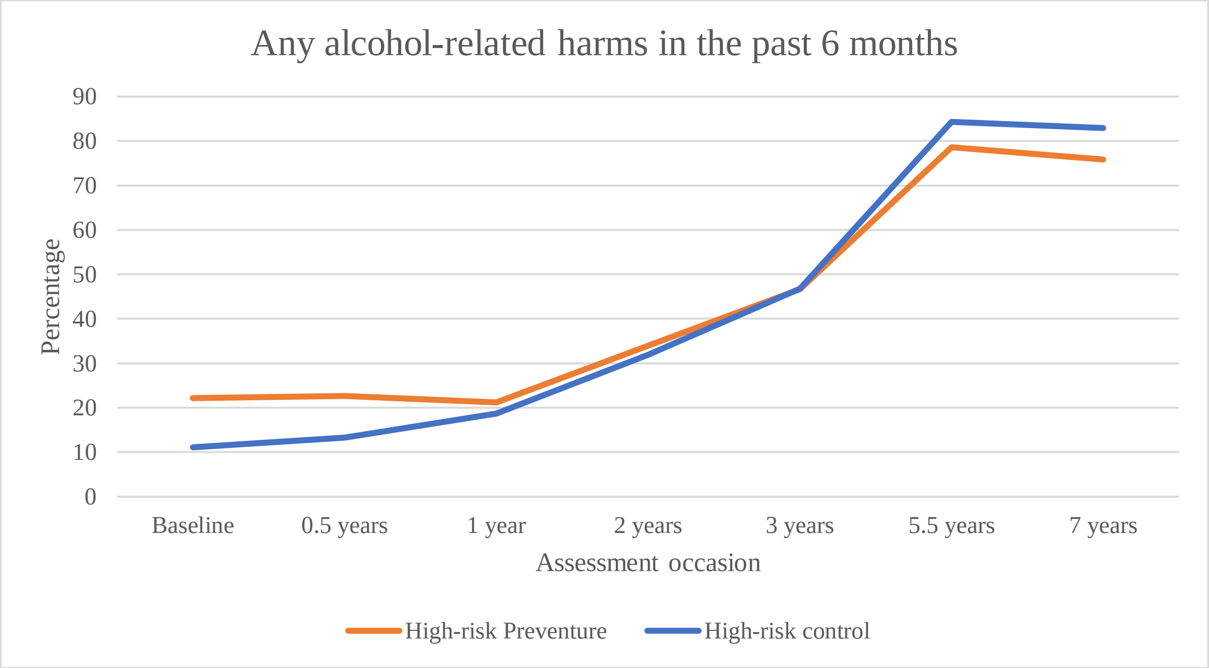

© 2022 Newton NC et al. *JAMA Network Open*.

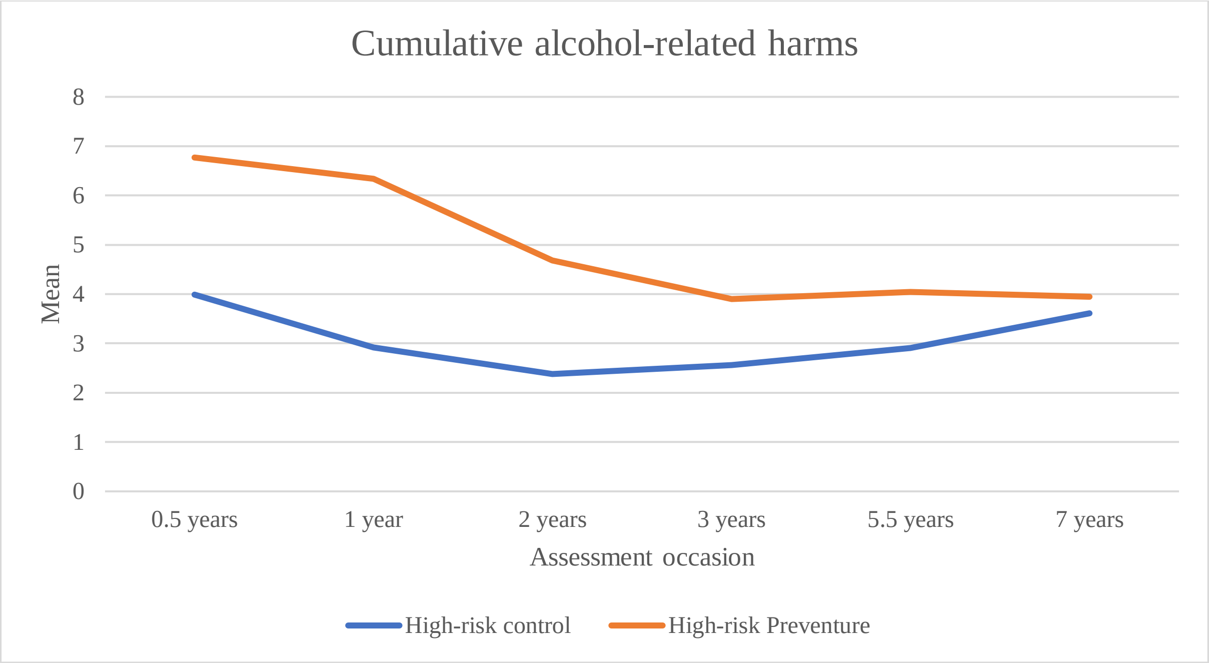

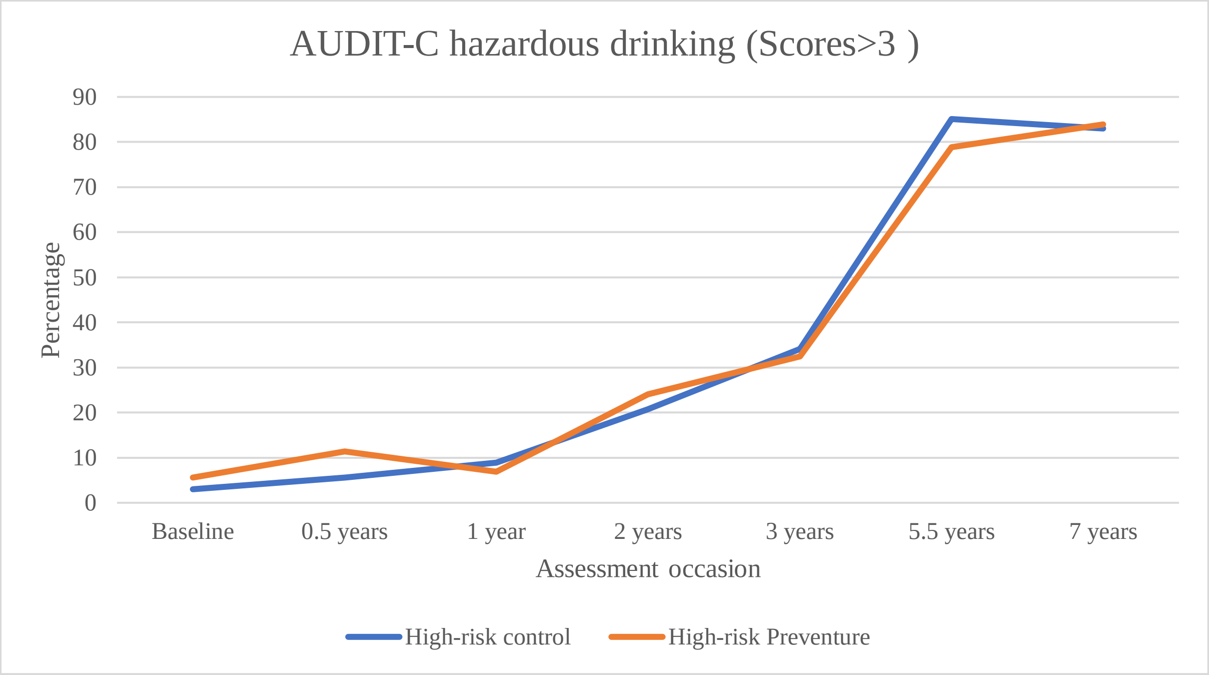

© 2022 Newton NC et al. *JAMA Network Open*.

eAppendix 2. Attrition Analysis

Table 1. Baseline Differences Between Those Lost to Follow-up and Those Present at the Long-term Follow-up

**Characteristic**	**OR**	**95% CI**
Sex [a]	1.80	1.23-2.67
Trial Group [b]	1.35	1.11-1.64
Monthly binge drinking	0.93	0.74-1.17
Hazardous alcohol use	0.93	0.74-1.16
Alcohol-related harm	0.96	0.78-1.17
**Personality trait**		
Impulsivity	0.90	0.59-1.41
Sensation Seeking	1.10	0.70-1.64
Anxiety sensitivity	0.95	0.61-1.46
Negative thinking	1.10	0.68-1.71
	**B**	**95% CI**
Frequency of alcohol-related harm	0.11	-0.09-0.31

[a] Reference group females; [b] Reference group control.

© 2022 Newton NC et al. *JAMA Network Open*.

eAppendix 3. Sensitivity Analyses

To account for baseline differences in sex, sex was included as a covariate in the models and adjusted estimates are presented in Table 2. Comparisons between those absent and present at long-term follow-up are presented in Table 3.

Table 2. Relative Change in Log Odds of Alcohol Outcomes at 7 Years Post Baseline (Adjusted for Baseline Differences in Sex)

	**OR**	**95% CI**	***p***
Monthly binge drinking			
Interaction effects			
Preventure × time	0.80	0.56-1.13	0.20
Preventure × time^2	1.03	0.98-1.08	0.27
Any alcohol-related harm			
Interaction effects			
Preventure × time	0.81	0.70-0.94	0.00
Hazardous alcohol use			
Interaction effects			
Preventure × time	0.91	0.63-1.31	0.60
	B	**95% CI**	***p***
Frequency of alcohol-related harm (total RAPI)			
Preventure × time	-0.14	-0.27 - -0.01	0.04

To examine the potential ceiling effect of alcohol outcomes around the 5.5-year survey assessment when participants are legally able to purchase alcohol and use becomes normative, we re-constructed the models without the 7-year survey assessment (see Table 3). All secondary models include a three-level hierarchical structure, with random intercepts at both the school and individual level. The better fitting continuous growth model for time was applied to all variables, where time represents the relative 1-year change in the odds of binge drinking, any alcohol-related harm and hazardous alcohol use and the relative 1-year change in the mean frequency of alcohol-related harm for the intervention group compared to control.

© 2022 Newton NC et al. *JAMA Network Open*.

Table 3. Relative Change in Odds and Mean Frequency of Alcohol Outcomes and Mean Frequency of Alcohol Harms From Baseline to 5.5 Years

	**OR**	**95% CI**	***p***
Monthly binge drinking			
Preventure × time (5.5 years)	0.87	0.77-0.99	0.04
Any alcohol-related harm			
Preventure × time (5.5 years)	0.79	0.68-0.93	0.01
Hazardous alcohol use (AUDIT-C)			
Preventure × time (5.5 years)	0.91	0.84-0.99	0.03
	B	**95% CI**	***p***
Frequency of alcohol-related harms			
Preventure × time (5.5 years)	-0.21	-0.29 - -0.12	0.00

© 2022 Newton NC et al. *JAMA Network Open*.
