# Supplementary material for: Effect of Selective Personality-Targeted Alcohol Use Prevention on 7-Year Alcohol-Related Outcomes Among High-risk Adolescents: A Secondary Analysis of a Cluster Randomized Clinical Trial
Source: JAMA Netw Open. 2022 Nov 17;5(11):e2242544. doi: 10.1001/jamanetworkopen.2022.42544 (PMC9672969; doi:10.1001/jamanetworkopen.2022.42544)
Supplement: Supplement 3. — Data Sharing Statement [file jamanetwopen-e2242544-s003.pdf]

Data Sharing Statement

Newton. Effect of Selective Personality-Targeted Alcohol Use Prevention on 7-Year Alcohol-Related Outcomes Among High-Risk Adolescents. *JAMA Netw Open*. Published November 17, 2022. doi:10.1001/jamanetworkopen.2022.42544

Data
Data available: No

Additional Information
Explanation for why data not available: Data would be made available upon request and will require ethical approval.
